# Assessment of metagenomic workflows using a newly constructed human gut microbiome mock community

**DOI:** 10.1093/dnares/dsad010

**Published:** 2023-05-31

**Authors:** Hiroshi Mori, Tamotsu Kato, Hiroaki Ozawa, Mitsuo Sakamoto, Takumi Murakami, Todd D Taylor, Atsushi Toyoda, Moriya Ohkuma, Ken Kurokawa, Hiroshi Ohno

**Affiliations:** Advanced Genomics Center, National Institute of Genetics, 1111 Yata, Mishima, Shizuoka 411-8540, Japan; Department of Informatics, National Institute of Genetics, 1111 Yata, Mishima, Shizuoka 411-8540, Japan; Laboratory for Intestinal Ecosystem, RIKEN Center for Integrative Medical Sciences, 1-7-22 Suehiro-cho, Tsurumi-ku, Yokohama, Kanagawa 230-0045, Japan; Immunobiology Laboratory, Graduate School of Medical Life Science, Yokohama City University, 1-7-29 Suehiro-cho, Tsurumi-ku, Yokohama, Kanagawa 230-0045, Japan; Laboratory for Intestinal Ecosystem, RIKEN Center for Integrative Medical Sciences, 1-7-22 Suehiro-cho, Tsurumi-ku, Yokohama, Kanagawa 230-0045, Japan; Immunobiology Laboratory, Graduate School of Medical Life Science, Yokohama City University, 1-7-29 Suehiro-cho, Tsurumi-ku, Yokohama, Kanagawa 230-0045, Japan; Microbe Division / Japan Collection of Microorganisms, RIKEN BioResource Research Center, 3-1-1 Koyadai, Tsukuba, Ibaraki 305-0074, Japan; Advanced Genomics Center, National Institute of Genetics, 1111 Yata, Mishima, Shizuoka 411-8540, Japan; Department of Informatics, National Institute of Genetics, 1111 Yata, Mishima, Shizuoka 411-8540, Japan; Laboratory for Microbiome Sciences, RIKEN Center for Integrative Medical Sciences, 1-7-22 Suehiro-cho, Tsurumi-ku, Yokohama, Kanagawa 230-0045, Japan; Advanced Genomics Center, National Institute of Genetics, 1111 Yata, Mishima, Shizuoka 411-8540, Japan; Department of Genomics and Evolutionary Biology, National Institute of Genetics, 1111 Yata, Mishima, Shizuoka 411-8540, Japan; Microbe Division / Japan Collection of Microorganisms, RIKEN BioResource Research Center, 3-1-1 Koyadai, Tsukuba, Ibaraki 305-0074, Japan; Advanced Genomics Center, National Institute of Genetics, 1111 Yata, Mishima, Shizuoka 411-8540, Japan; Department of Informatics, National Institute of Genetics, 1111 Yata, Mishima, Shizuoka 411-8540, Japan; Laboratory for Intestinal Ecosystem, RIKEN Center for Integrative Medical Sciences, 1-7-22 Suehiro-cho, Tsurumi-ku, Yokohama, Kanagawa 230-0045, Japan; Immunobiology Laboratory, Graduate School of Medical Life Science, Yokohama City University, 1-7-29 Suehiro-cho, Tsurumi-ku, Yokohama, Kanagawa 230-0045, Japan; Laboratory for Microbiome Sciences, RIKEN Center for Integrative Medical Sciences, 1-7-22 Suehiro-cho, Tsurumi-ku, Yokohama, Kanagawa 230-0045, Japan

**Keywords:** human gut microbiome, bacterial mock community, metagenomics, 16S rRNA gene sequencing

## Abstract

To quantify the biases introduced during human gut microbiome studies, analyzing an artificial mock community as the reference microbiome is indispensable. However, there are still limited resources for a mock community which well represents the human gut microbiome. Here, we constructed a novel mock community comprising the type strains of 18 major bacterial species in the human gut and assessed the influence of experimental and bioinformatics procedures on the 16S rRNA gene and shotgun metagenomic sequencing. We found that DNA extraction methods greatly affected the DNA yields and taxonomic composition of sequenced reads, and that some of the commonly used primers for 16S rRNA genes were prone to underestimate the abundance of some gut commensal taxa such as *Erysipelotrichia*, *Verrucomicrobiota* and *Methanobacteriota*. Binning of the assembled contigs of shotgun metagenomic sequences by MetaBAT2 produced phylogenetically consistent, less-contaminated bins with varied completeness. The ensemble approach of multiple binning tools by MetaWRAP can improve completeness but sometimes increases the contamination rate. Our benchmark study provides an important foundation for the interpretation of human gut microbiome data by providing means for standardization among gut microbiome data obtained with different methodologies and will facilitate further development of analytical methods.

## 1. Introduction

Understanding the human gut microbiome is important for human health and disease, and it is being studied intensively.^1^ To investigate the composition of the human gut microbiome, 16S rRNA gene amplicon sequencing and shotgun metagenomic sequencing are being frequently applied. Because the human gut microbiome is highly variable among individuals, large sample sizes are necessary to obtain robust results.^2^ Therefore, several meta-analyses, which integrate sequence data from multiple human gut microbiome studies, have been conducted.^3,4^ However, when comparing data across multiple studies, one must be aware of method-specific biases which may be introduced at any step during wet-lab (e.g., sample preservation, DNA extraction, PCR amplification, and sequencing) as well as *in silico* experiments.^5–7^ On the other hand, methods for microbiome analyses are still developing. Indeed, more than half (11 of the 21) of DNA extraction protocols examined by Costea *et al.*^5^ are already unavailable due to the discontinuation of the extraction kits. Because the bioinformatics tool algorithms and bacterial taxonomic names are frequently changing, it has also become difficult to compare taxonomic and functional composition results analyzed using different versions of software or reference databases. Furthermore, since taxonomic and functional analyses at higher resolution have become possible with the development of sequencing technology (e.g., sequencing quantity and quality improvements, long-read sequencing technologies), continuous assessments of biases in microbiome analyses are fundamental for the appropriate understanding of human gut microbiome data.

One of the biggest hurdles for direct comparison of data obtained with different methodologies is the lack of measures for standardization since the gut microbial compositions are unknown, making it extremely difficult to reach ‘the correct answer’ for the quantification of gut microbiome data when using faecal samples. To circumvent this and achieve the bridging of multiple gut microbiota data comparisons, a ‘mock community’, which is an artificially constructed microbiome with defined composition, of human gut microbiota is indispensable; it will enable the assessment of potential biases introduced with each method and the compensation for biases among the data.^8,9^ Currently, the Human Microbiome Project-designed American Type Culture Collection (ATCC) mock community (HMP-ATCC mock)^10^ and ZymoBIOMICS mock community are commercially available and commonly used for research.^11,12^ However, the use of these mock communities may not be the best choice for human gut microbiome studies because they do not contain the major bacterial taxa of typical human gut microbiota. In addition, the HMP-ATCC mock is categorized as Biosafety level 2 (BSL2) because it contains pathogens such as *Neisseria meningitidis* and *Yersinia enterocolitica*. Recently, some human microbiome-specific mock communities were developed and are commercially available [e.g., ZymoBIOMICS Gut Microbiome Standard and NBRC (National Institute of Technology and Evaluation Biological Resource Center) Human Microbial Cell Cocktail]. These human microbiome-specific mock communities have different taxonomic characteristics. ZymoBIOMICS Gut Microbiome Standard contains a broad range of taxa (i.e., *Bacteria*, *Archaea* and *Eukaryota*), including multiple *Escherichia coli* strains. NBRC Human Microbial Cell Cocktail contains major bacterial taxa of typical human gut microbiota, some non-human origin taxa (*Pseudomonas putida* and *Lactobacillus delbrueckii*), and major human skin taxa (*Staphylococcus epidermidis* and *Cutibacterium acnes*).^13^ Since the studies using mock communities have a variety of purposes and analytical methods, providing multiple choices of mock community resources for researchers is desirable.

Here, we constructed a new mock community which represents the major taxa in the human gut bacterial community. Using this mock community and an originally established qPCR-based bacterial quantification method with each strain-specific primer set, we compared the performance of 10 commonly used DNA extraction methods. Then, we conducted short-read based 16S rRNA gene amplicon and shotgun metagenomic sequencing to assess the influence of seven PCR primer pairs for amplicon sequencing, as well as four types of bioinformatics analysis strategies to assess the inference of taxonomic composition. We also compared the performance of two binning tools (i) MetaBAT2,^14^ one of the most popular tools for metagenomic binning to reconstruct genomes from metagenomic data and (ii) MetaWRAP,^15^ a commonly used wrapper tool integrating three metagenomic binning tools.

## 2. Materials and methods

### 2.1 Mock community design

**Figure 1.**
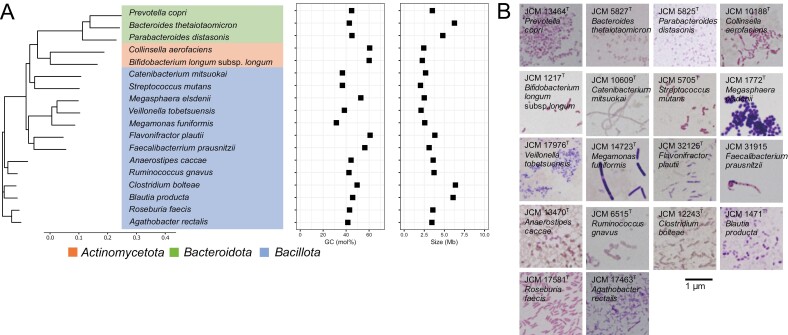
**Construction of mock bacteria community mimicking human faeces.** (A) 18 bacterial strains were used for the mock community in this study. Right panel: GC content and genome size of each bacterium are shown. (B) Gram staining images of the 18 strains.

To design the mock community, we considered three points: (i) habitat, (ii) usability and (iii) taxa balance. We chose type strains of 18 bacterial species (Fig. 1A and Supplementary Table S1) based on the criteria that (i) they are major bacterial species in healthy human faeces regardless of country/residential area (Supplementary Table S2 and Nishijima *et al*.^16^), (ii) belong to BSL1, are cultivable, and can be obtained without restriction, and that their whole genomes have been sequenced. In addition, (iii) we avoided including multiple species from a genus in the mock community to reduce the taxonomic bias of the mock community. They belong to distinct genera of phyla *Bacillota*, *Bacteroidota* or *Actinomycetota*. The molecular weight of the genome for each strain was estimated based on the genome length and GC content.

### 2.2 Gram staining and microscopy

Gram staining was performed as described in the Supplementary methods. After completely drying the slides, the cells of the 18 bacterial strains were observed under a BX51 microscope (Olympus Corporation, Tokyo, Japan).

### 2.3 Flow cytometry-based bacterial cell count

The number of cells for the 18 strains was counted using the Bacteria Counting Kit for flow cytometry (Thermo Fisher Scientific, Waltham, MA, USA). Some bacterial suspensions were subjected to ultrasonication (68 kHz for 20 sec x 10–30 cycles, HZ-380, As ONE Corporation, Osaka, Japan) to disrupt cellular chains/aggregates. Diluted bacterial culture was stained using the LIVE/DEAD™/Bac/Light™ Bacterial Viability and Counting Kit for flow cytometry (Thermo Fisher Scientific) for 5 minutes. Stained bacterial cells were counted by a BD FACS Canto II (BD Biosciences, Franklin Lakes, NJ, USA). Then, 18 bacterial strains were mixed so that the cell number of each strain was equal (5.0 x 10^7^ cells/ ml).

### 2.4 DNA extraction

Ten DNA extraction methods were evaluated (Supplementary Table S3 and Supplementary methods). After considering the extracted DNA quantities and qualities, six DNA extraction methods (i.e., Enzyme, HMP, MetaHIT, BeadsPhenol, PureLink and Zymo; Supplementary Table S3) were subsequently used for sequence-based taxonomic composition evaluation. The extracted DNAs were stored at -20°C until PCR and sequencing.

### 2.5 Quantitative real-time PCR (qPCR) measurement

To estimate the absolute abundance of each strain in the mock community sample, strain-specific qPCR primer pairs targeting the RNA polymerase subunit beta gene (*rpoB*) were designed using the Primer3 web application^17^ (Supplementary Table S4). The details of the qPCR procedure are described in the Supplementary methods.

### 2.6 16S rRNA gene amplification and amplicon sequencing

We selected seven commonly used prokaryotic universal primer pairs for amplicon sequencing analysis of the 16S rRNA genes of the mock community samples (Supplementary Table S5). Sequencing libraries were constructed using the two-step PCR amplification protocol provided by Illumina^18^ (Supplementary methods). After the purification, the amplicon libraries were analyzed on an Illumina MiSeq with 350 bp (forward, Read1) + 250 bp (reverse, Read 2) paired-end sequencing. The reason for this MiSeq run setting is that the 3’ end of Read 2 (250-300 bp region) is often of extremely low quality, so the number of cycles for Read 1 was increased to avoid producing low-quality reads.

### 2.7 Shotgun metagenomic sequencing

For shotgun sequencing, genomic DNA was fragmented to an average size of 500 bp with the DNA Shearing System M220 (Covaris, Wobum, MA, USA). To remove amplification bias associated with PCR,^13,19^ sequencing libraries were constructed using a TruSeq DNA PCR-Free Library Prep kit (Illumina, San Diego, CA, USA) and were size-selected on an agarose gel using a Zymoclean Large Fragment DNA Recovery Kit (Zymo Research, Orange, CA, USA). The constructed libraries were sequenced on an Illumina HiSeq 2500 using a Rapid mode chemistry or an Illumina NovaSeq 6000 using an SP Reagent Kit with 250 bp paired-end sequencing. All 16S rRNA and shotgun sequence data are available from DDBJ DRA under BioProject ID PRJDB10817.

### 2.8 Inference of the strain composition based on 16S rRNA gene amplicons

The paired-end reads from four amplicon sequencing libraries (341F-785R, 342F-806RS, 515FY-926RY and 968F-1390R) were merged using NGmerge version 0.2.^20^ Because the amplicon lengths of three primer pairs (27Fmod-338R, 515F-806R and 515FY-806RN) were less than or almost equal to the forward read length (350 bp), only the forward reads were used for the bioinformatics analyses. To simultaneously assess the strain compositions of both amplicon and shotgun metagenomic sequencing, we used MAPseq version 1.2.5, which is a taxonomic classification tool for short reads based on the DNA sequence similarity,^21^ with the 16S rRNA gene sequences of corresponding strains as the reference database. The threshold of species assignment in the top hit result of the MAPseq search was an identity > 97% and an alignment length > 100 bp. To validate the results of MAPseq, the amplicon sequencing data was also analyzed by DADA2 version 1.14,^22^ a denoising and dereplication tool for the amplicon sequences that is commonly used in many workflows (e.g., the default parameters of QIIME2 use DADA2 for the sequence denoising), and we confirmed the consistency between the results from these two tools (Pearson correlation coefficient of taxonomic compositions = 0.84–0.99, Supplementary Table S6). Inferred abundance of each strain was normalized by genome size and 16S rRNA gene copy number of the corresponding strain (Supplementary Table S1).

Taxonomic coverage of the primer-matched 16S rRNA gene sequences in the Ribosomal Database Project was calculated by ProbeMatch web tool with the following parameters: Strain = Both, Source = Both, Size ≥ 1,200, Quality = Good, and within 1 mismatch.^23,24^ We did not conduct ProbeMatch survey for the 27Fmod and 1390R because they are one of the most frequently used primer pairs to amplify near full-length 16S rRNA gene sequences and thus 16S rRNA gene sequences deposited in the public database are reportedly biased to match this primer pair.^25^

### 2.9 Inference of strain composition based on shotgun metagenomic sequences

**Figure 2.**
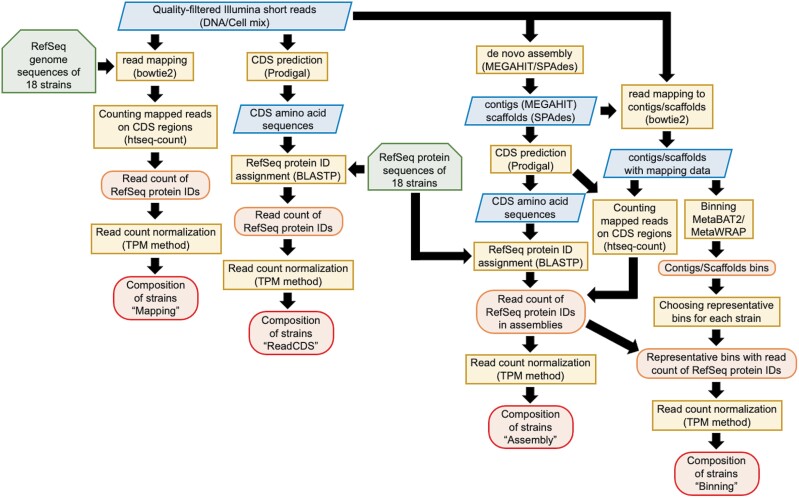
**Procedures to assign metagenomic sequences to the corresponding protein-coding sequences (CDSs) of the strains.** The flow chart is shown to align the sequence reads to CDSs of the 18 strains which were annotated with RefSeq protein IDs, parallelograms, sequence data; hexagons, sequence database; rectangles, analysis; ellipses, output data.

Short read pairs were quality filtered by using fastp version 0.20^26^ with parameters ‘-n 1 -l 70’. To assess the sequenced read abundance of the 18 strains in each metagenomic sample, the reads were aligned to the protein-coding sequences (CDSs) of the 18 strains which were annotated with RefSeq protein IDs, and enumerated with four different strategies: short read mapping (i) on the reference genomes (Mapping), (ii) on whole contigs/scaffolds de novo assembled by MEGAHIT version 1.2.9^27^ and SPAdes version 3.13.1 with the metagenomic mode^28^ (Assembly), or (iii) on contigs/scaffolds retrieved within the bins by MetaBAT2 (Binning), and (iv) counting the CDS fragments predicted from the unassembled short reads (ReadCDS) by Prodigal version 2.6.3 with the metagenomic mode^29^ (Fig. 2). Although it is possible to search DNA sequences from reads directly against amino acid sequences with BLASTX, DIAMOND, etc., these tools internally extract open reading frames (ORFs) and translate them into amino acid sequences. In this case, the original amino acid sequences cannot be easily obtained, which is inconvenient for later evaluation of the results. Therefore, in this study, Prodigal-based metagenomic gene prediction was performed from reads, and the predicted amino acid sequences are used for analysis. Short reads were mapped on the reference genomes, contigs, and scaffolds by bowtie2 version 2.3.5.1,^30^ and aligned reads within CDS regions were enumerated by htseq-count version 0.11.2 with the union mode.^31^ Detailed procedures are described in the Supplementary methods. Identical CDSs with the same RefSeq protein IDs among the 18 strains were removed from the reference database, and only CDSs having unique RefSeq protein IDs were considered (Supplementary Table S1).

Read counts of each CDS enumerated by the four respective strategies were then normalized based on the length of the CDS and the total count of enumerated reads in a sample as in the transcript per million (TPM) method.^32^ Median values of normalized read counts of all examined CDSs or 31 single-copy genes (Supplementary Table S7) within each strain were used for the representation of the abundance of that strain. In addition to the manual inference described above, the taxonomic composition of each sample was also assessed using MetaPhlAn2 version 2.96.1.^33^

### 2.10 Statistics

Statistical analyses were conducted in an R version 3.6.0 platform. Shannon and Simpson diversity indices were calculated with the vegan version 2.5.6 package to evaluate the evenness of the strain compositions. A principal component analysis based on the relative abundance of strain compositions was performed with the prcomp function.

## 3. Results and discussion

### 3.1 Mock community construction

To objectively compare and evaluate DNA extraction methods for gut microbiome analysis, we aimed to construct a mock community representing human faecal microbiota by mixing an equal amount of cells of 18 selected strains (Cell-mix sample). Seven of them are Gram-negative and the other eleven are Gram-positive.

It is often difficult to construct a mock community of the desired cell ratio.^34^ Indeed, cell morphologies of the 18 strains vary from 0.2-µm-diameter cocci to 2.0-µm-long bacilli (Fig. 1B), making the estimation of cell concentration by densitometer difficult. For a more accurate estimation of cell concentration, we employed flow cytometry-based bacterial cell counting. Initial trials suggested that flow cytometry underestimated the cell counts of bacteria that form cellular chains or aggregates under Gram staining (i.e., *Megasphaera elsdenii* and *Faecalibacterium prausnitzii*; Fig. 1B). To improve counting accuracy, ultrasonication was applied to the cell suspensions to disrupt cellular chains or aggregates. Consequently, the counted cell numbers of the *Megasphaera elsdenii* and *Faecalibacterium prausnitzii* strains increased two- to four-fold (Supplementary Fig. S1). On the other hand, the counts of the *Catenibacterium mitsuokai*, *Megamonas funiformis* and *Anaerostipes caccae* strains showed a decreasing tendency (Supplementary Fig. S1). We, therefore, decided to sonicate only two strains (*Megasphaera elsdenii* JCM 1772 and *Faecalibacterium prausnitzii* JCM 31915) which obviously showed an increase in cell count using ultrasonication. Since faecal bacteria in nature are a mixture of both live and dead cells, we counted all bacterial dots regardless of their viability.

We also checked for bacterial contamination in the buffers that were used for the bacterial count, and only the FACS buffer contained a small number of bacteria according to our FACS quantification, representing only ~0.5% (0.3% in 0.85% NaCl buffer) of the total beads count used for measurement.

Our mock community has an appropriate balance of taxa for the method evaluation purposes. Three major phyla of the human gut microbiome are included, evenly mixed, no multiple species of the same genus, no extremely phylogenetically distinct taxon (i.e., *Eukaryota* and *Archaea*) and no obvious contamination. This appropriate balance of taxa from our mock community is important to evaluate the baseline performance of wet and dry methods for human gut microbiome analysis.

### 3.2 Optimization of qPCR using the rpoB primers for quantification

We designed strain-specific primers targeting *rpoB*, a single-copy gene conserved within the domain *Bacteria*^35^ (Supplementary Table S4), and conducted qPCR for accurately estimating the abundance of each strain. Before the quantification, we performed qPCR under several different conditions (Supplementary Table S8) and confirmed that the amplification efficiency of the DNA template of each of the 18 strains fell in the range of 90%~100% for at least one condition (Supplementary Fig. S2A). We then confirmed the specificity of the qPCR by using a DNA template which contains an equal amount of genomic DNA for all 18 strains (DNA-mix sample). The relative abundance of each strain within the DNA-mix sample estimated by these qPCR analyses was nearly equivalent to the theoretical value (Supplementary Fig. S2B); therefore, we concluded that qPCR using the *rpoB* primer set is adequate for estimating the composition of bacteria in the DNA mixture.

### 3.3 Comparison of DNA quantity and quality among DNA extraction methods

**Figure 3.**
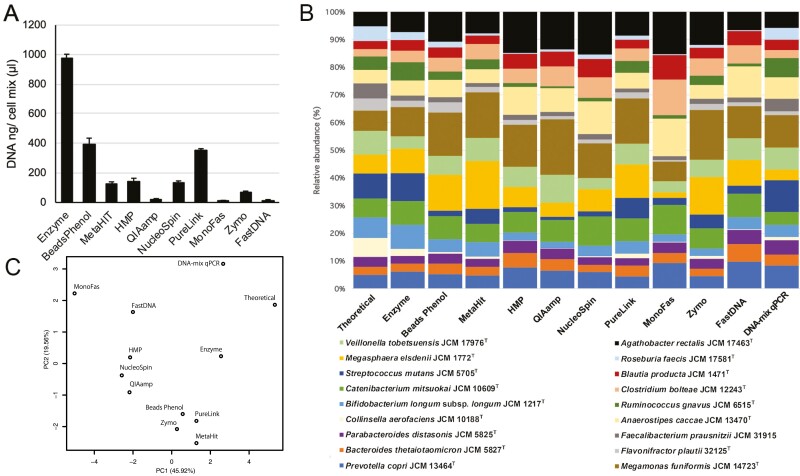
**Influence of DNA extraction method on DNA yields and bacterial composition.** (A) Amount of DNA yields and (B) composition of the 18 strains in the Cell-mix samples extracted with 10 different DNA extraction methods. The proportion of each strain was estimated from the results of qPCR using the *rpoB* primer. The DNA-mix sample contains an equal amount of genomic DNA for all 18 strains. The DNA-mix qPCR abundance is the qPCR quantified result of the DNA-mix sample on these 18 strains using each strain-specific *rpoB* qPCR primer pair. The theoretical abundance was estimated from the genome length and GC content of each strain when the amount of DNA in the DNA-mix sample was equal among 18 strains. (C) The principal component analysis (PCA) score plot of the composition of the 18 bacteria using 10 different DNA extraction methods.

We compared the efficiency of 10 different commonly used DNA extraction methods with our Cell-mix samples (Supplementary Table S3). In our hands, the enzyme method gave the highest DNA yield followed by the PureLink kit and the Beads-Phenol method (Fig. 3A). qPCR quantification of *rpoB* genes indicated a notable difference in the relative abundance of the 18 strains among the extraction methods (Fig. 3B). The principal component analysis result of the taxonomic composition suggested that the enzyme method could extract DNA from the Cell-mix more efficiently and that its strain composition was closest to that of the theoretical calculation and DNA-mix sample compared to the other extraction methods (Fig. 3C). In addition, Supplementary Fig. S3 shows that the DNA yields of the enzyme methods were the highest among the 10 methods when DNA was separately extracted from each of the 18 strains, suggesting that the enzyme method is the most suitable for extracting DNA from a wide variety of bacteria existing in the human gut. We also checked the DNA purity by the ratio of absorbance (nm) at 260/280 and 260/230 (Supplementary Table S9). The results suggested that some extraction methods have protein and other contaminant issues. Overall, these results indicate that the efficiency of DNA extraction varies by strains and that the enzyme method gave the best extraction results in our hands. Based on these observations, we selected 6 out of 10 tested methods for further sequencing-based comparisons (Supplementary Table S3).

### 3.4 Influence of the 16S rRNA gene primers on the taxonomic composition

**Figure 4.**
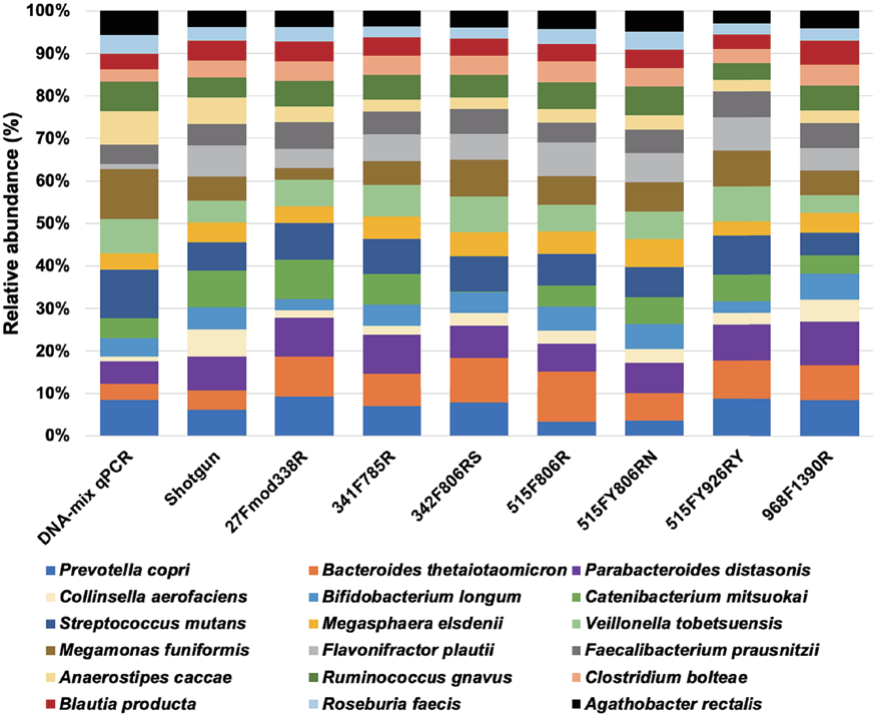
**Strain compositions of the amplicon sequencing data.** Strain compositions of the DNA-mix sample were inferred by conducting qPCR, amplicon sequencing with seven different primer pairs, and shotgun metagenomic sequencing.

We obtained an average of 96000 pairs of 16S rRNA gene reads from the DNA-mix sample amplified with seven different primer pairs (Supplementary Tables S5 and S10). Inferred taxonomic compositions from the 16S rRNA gene amplicon sequencing were mostly consistent among the different primer pairs with a notable exception that the 342F-806RS primer pair only weakly amplified the 16S rRNA genes of *Catenibacterium mitsuokai* (*Bacillota*, *Erysipelotrichia*) (Fig. 4). Indeed, although the 342F-806RS and 341F-785R primer pairs amplified almost identical regions, amplification efficiency for *Ca. mitsuokai* was drastically different between these primer pairs. We found that two single-base mismatches exist between the 806RS primer sequence and the 16S rRNA gene sequences of *Ca. mitsuokai*. In addition, the *in silico* comparison between the primers and 16S rRNA gene sequences deposited in the Ribosomal Database Project indicated that the 806RS primer sequence matches *Bacillota* (Supplementary Table S11), but does not match *Erysipelotrichia* sequences. Such inefficiency of 342F-806RS against 16S rRNA genes of *Erysipelotrichia* is probably attributable to the absence of ambiguous nucleotides in the 342F and 806RS primer sequences (Supplementary Table S5). The 342F-806RS primer pair had been designed to specifically amplify prokaryotic rRNA genes and minimize the non-targeted amplification of eukaryotic rRNA genes.^25^ The human gut (faeces) contains a relatively low amount of eukaryotic DNA^36^; thus, the use of the 342F-806RS primer pair should be avoided. The other six primer pairs did not exhibit notable biases in the inferred taxonomic compositions (Fig. 4); however, *in silico* survey indicated that the 338R and 968F primers contain more than two single-base mismatches against the 16S rRNA gene sequences of most taxa in the phyla *Verrucomicrobiota* (338R) and *Methanobacteriota* (338R and 968R). These two phyla contain minor but important taxa in the human gut such as *Akkermansia muciniphila* and *Methanobrevibacter smithii*, respectively^37,38^ (Supplementary Table S11). Therefore, the 341F-785R, 515F-806R, 515FY-806RN and 515FY-926RY primer pairs are likely to be more suitable for human gut microbiome analysis.

Although the strain compositions were mostly consistent among the different primer sets, they were significantly different from those inferred from shotgun metagenomic sequencing (Kruskal–Wallis test, *P* < 0.001) (Fig. 4 and Supplementary Fig. S4). The relative abundance of *Bacteroides thetaiotaomicron* was enriched in all amplicon samples compared to the shotgun metagenomic sample (average fold change: 2.0). On the other hand, the relative abundances of *Anaerostipes caccae* and *Collinsella aerofaciens* were under-represented in all amplicon samples compared to the shotgun metagenomic sample (average fold change: 0.5 and 0.4, respectively). The relative abundances of other strains also varied between each amplicon sample and the shotgun metagenomic sample, but we could only observe consistent trends in these three strains. Although the taxonomic assignment methods are identical between the amplicon and shotgun metagenomic sequencing data of the DNA-mix sample, there are several artificial biases that can be recruited (e.g., PCR bias, PCR chimaera frequencies, GC bias, bias associated with the sequence library preparation kit, taxonomic assignment difficulties of partial sequences, etc.). For example, previous studies suggested that the GC content of genomes affects the taxonomic abundance of shotgun metagenomic sequence data.^13,39^ However, we did not find any correlation between the taxonomic abundance of shotgun metagenomic sequencing data of the DNA-mix sample and the GC content of each genome (Pearson correlation coefficient = −0.01). There may be some other artificial biases that lead to the taxonomic composition differences among amplicon and shotgun metagenomic data.

### 3.5 Assembly of shotgun metagenomic sequences

We next aimed to assemble all obtained shotgun metagenomes by using SPAdes and MEGAHIT, respectively. We obtained 4 million and 29 million quality-filtered read pairs using the HiSeq 2500 and NovaSeq 6000 sequencers, respectively, for a DNA-mix sample, and on average 25 million quality-filtered read pairs for six Cell-mix samples (Supplementary Table S12). Compared within the same sample, scaffolds generated by SPAdes exhibited greater maximum length and N50 values than those of MEGAHIT contigs (Supplementary Table S12).

### 3.6 Strain composition of the shotgun metagenomic sequencing samples

Since the beginning of the metagenomic era, there has been an ongoing debate about which bioinformatics strategies should be used to best estimate taxonomic compositions for shotgun metagenomics data.^40,41^ For microbial communities such as soils, which have high phylogenetic diversity and contain closely related strains, CDS prediction from unassembled short reads is frequently conducted because it is often difficult to construct high-quality contigs by the assembly.^42,43^ For microbial communities such as the human gut microbiome, where abundant sequenced genomes are available, the read mapping strategy is frequently applied for analyzing large numbers of samples at higher speed with fewer computer resources.^36,44^

**Figure 5.**
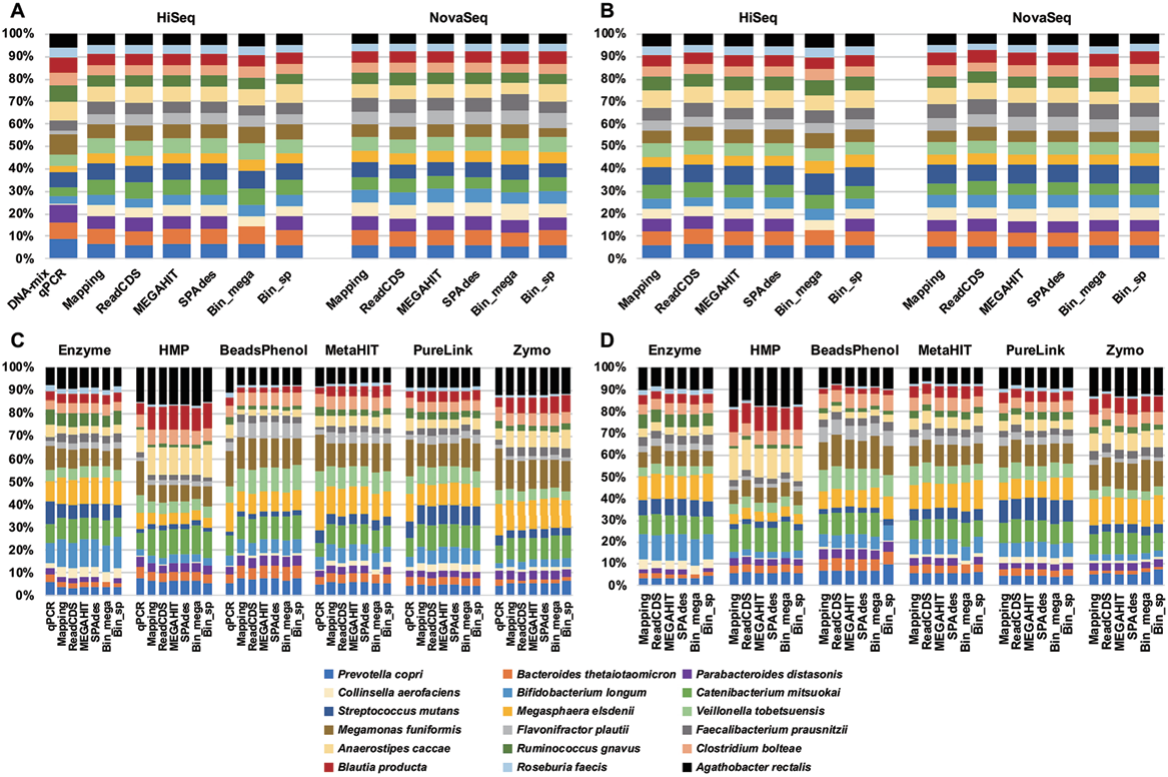
**Strain compositions of the shotgun metagenomic sequencing data assessed by the different bioinformatics analysis strategies.** The procedures of each strategy (i.e., Mapping, ReadCDS, Assembly and Binning) are explained in Methods and summarized in Fig. 2. ‘MEGAHIT’ and ‘Bin_mega’ use the MEGAHIT assembler in the metagenomic assembly. ‘SPAdes’ and ‘Bin_sp’ use the SPAdes assembler in the metagenomic assembly. ‘MEGAHIT’ and ‘SPAdes’ do not conduct the binning analysis. ‘Bin_mega’ and ‘Bin_sp’ conduct the binning analysis. Results from the DNA-mix sample (A and B) and the Cell-mix mock sample (C and D) are shown. Strain compositions were based on all examined CDSs (A and C) or 31 single-copy genes (B and D). For the DNA-mix sample, the read count of a CDS was additionally normalized based on the genome size of the corresponding strain after CDS-length normalization. The taxonomic composition of ‘DNA-mix qPCR’ is the qPCR quantification result of the DNA-mix sample (A). The taxonomic composition of ‘qPCR’ is the qPCR quantification result of the DNA extracted sample using each DNA extraction method (C).

**Figure 6.**
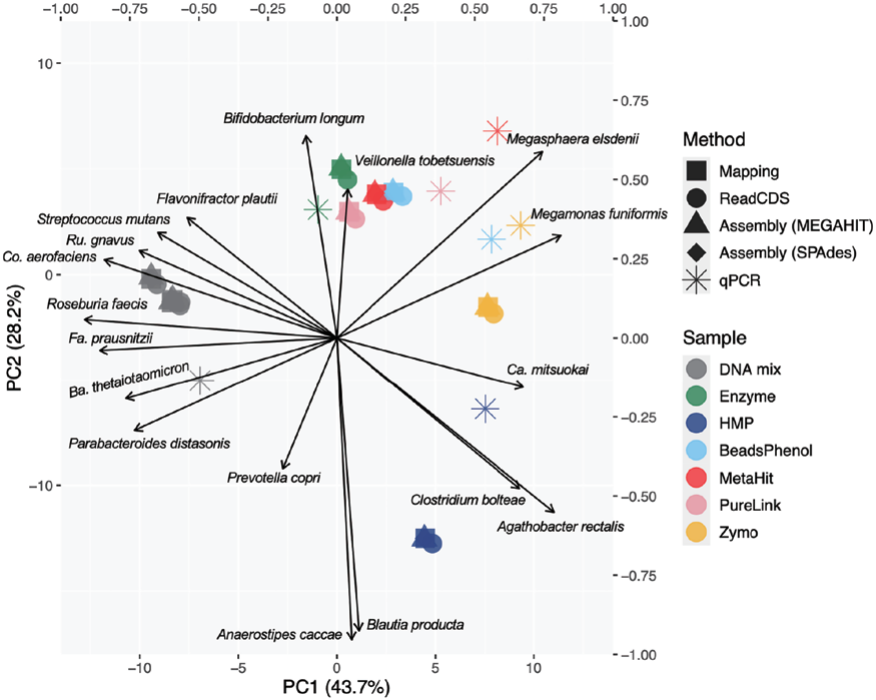
**Principal component analysis plot of the shotgun metagenomic samples.** The plot was based on the relative abundance of each strain. Results from the binning procedures were excluded. The arrows represent the loadings (correlation coefficients) between the principal component score and the abundance of the corresponding strain. Colours indicate the DNA extraction methods. Shapes of the points indicate the bioinformatics analysis strategies.

In this study, we inferred the strain compositions of each shotgun metagenomic sample by four different approaches: counting reads mapped on the protein-coding sequences within (i) the reference RefSeq genomes of 18 strains (Mapping), (ii) on *de novo* assembled contigs/scaffolds (Assembly), (iii) on contigs/scaffolds retrieved in the bins generated by MetaBAT2 (Binning) and (iv) predicting and counting CDSs from short reads (ReadCDS) (see Materials and methods, Supplementary methods and Fig. 2). The strain compositions based on the read counts of all examined CDSs or the 31 selected single-copy genes (Supplementary Table S7) showed almost no differences (Fig. 5), suggesting that when all reference genomes are available in a microbial community, counting the read abundance of single-copy genes is the most practical approach. There were also almost no differences among the strain assignment procedures of the Mapping, ReadCDS and Assembly strategies (Figs 5 and 6), while the Binning-based approach failed to reconstruct any bins for *Parabacteroides distasonis* from some assemblies such as MEGAHIT contigs of the DNA-mix sample sequenced by HiSeq (Fig. 5 and Supplementary Fig. S5). Thus, when appropriate reference genomes are available, Mapping, ReadCDS and Assembly strategies can precisely estimate taxonomic composition. We also note that the choice of short-read sequencers (Illumina HiSeq 2500 or NovaSeq 6000) had almost no influence on the results (Fig 5), suggesting that metagenomic sequencing data using these two sequencers are comparable.

Unlike the bioinformatics procedures and sequencing platforms, differences in DNA extraction methods substantially changed the sequenced read compositions of the strains as indicated by qPCR of *rpoB* (Fig. 3). Particularly, the sample extracted with the HMP method exhibited quite different strain composition from those of the other extraction methods or the DNA-mix sample; for example, the genomes of *Agathobacter rectalis*, *Anaerostipes caccae* and *Blautia producta* were more abundantly sequenced in the HMP sample than in the others, whereas reads of *Bifidobacterium longum*, *Collinsella aerofaciens* and *Roseburia faecis* were underrepresented in the HMP sample (Figs 5 and 6). Underestimation of Actinobacterial species by bead-beating methods was also reported in previous studies analyzing human feces^45^ and activated sludge.^46^ Community diversity indices also indicated that the strain composition for the HMP method was relatively skewed than for the other extraction methods (Supplementary Table S13); therefore, careful interpretation is necessary when comparing data obtained with different extraction methods. Except for the HMP standard protocol, it is difficult to conclude which DNA extraction method is more suitable as every DNA extraction method exhibited a similar, but not identical, taxonomic composition to the DNA-mix sample (Figs 5 and 6). Other studies indicated that the choice of the sequence library preparation kit is also the source of sequence data bias.^13,19^ We choose TruSeq PCR-free kit because the kit can avoid the PCR-associated amplification bias. We did not observe the typical bias in sequence data caused by sequence library preparation kits (e.g., the GC content-associated sequencing bias).

In addition to our manual assessment, the taxonomic composition of metagenomic samples was also assessed by MetaPhlAn2, a program pipeline commonly used for the inference of the taxonomic composition of metagenomes based on lineage-specific marker gene sets. MetaPhlAn2 reported the presence of almost all used species, and rarely assigned sequences to species unrelated to this study (Supplementary Fig. S6). The reads of *Bl. producta* JCM 1471^T^ were exceptionally misclassified as *Bl. coccoides* by MetaPhlAn2 (Supplementary Fig. S6). It is noted that one of the *Bl. coccoides* strains (strain NCTC 11035) in the MetaPhlAn2 reference database has 16S rRNA gene sequences of 99.87% identity with that of *Bl. producta* JCM 1471^T^, suggesting that these two strains are closely related and can easily be misclassified. Taxonomic compositions inferred by MetaPhlAn2 at both the species and genus level were different from those inferred by our manual assignment, or from the theoretical composition of the DNA-mix sample. Notably, MetaPhlAn2 was prone to underestimate the abundance of *Anaerostipes* and *Veillonella* (Supplementary Fig. S6).

Another commonly used taxonomic assignment method in the metagenomic analysis is a k-mer-based method. Kraken2 is a representative of the k-mer-based method for shotgun metagenomic analyses.^47^ Although the k-mer-based method is commonly used, the taxonomic assignment accuracy of the k-mer-based method is not sufficient to use species assignment in most cases.^48^ Therefore, we did not use the k-mer-based method in this study.

### 3.7 Completeness and contamination rates of the metagenomic bins

In each assembly, 77.0–90.2% of the total length of assembled contigs or scaffolds were clustered into 32–53 bins by MetaBAT2 (Supplementary Table S14). Completeness of the bins which most highly represented the original genomes (‘representative bins’; for definition, see Supplementary methods Section 5, (iii) ‘Binning’ varied among the strains. For example, representative bins of *Bacteroides thetaiotaomicron* from almost all assemblies boasted more than 90% completeness, while strains such as *Anaerostipes caccae*, *Prevotella copri* and *Faecalibacterium prausnitzii* exhibited less than 50% completeness for most of the assemblies (Supplementary Figs S5 and S7). We investigated the distribution of RefSeq ID-assigned CDSs within the contigs of all obtained bins and found that contigs of the strains such as *Prevotella copri* and *Faecalibacterium prausnitzii* were often split into multiple bins (Supplementary Fig. S8). Contamination was not observed in most of the representative bins, while strains such as *Bl. producta* and *Ruminococcus gnavus* exhibited relatively high contamination rates for a few assembly results (Supplementary Fig. S7). Collectively, MetaBAT2 is more likely to report phylogenetically consistent bins with varied completeness among the examined strains.

We also evaluated the ensemble approach binning tool MetaWRAP for the assembled sequence data. MetaWRAP first individually conducts binning using three tools MetaBAT2, MaxBin2^49^ and CONCOCT.^50^ Then, MetaWRAP merges the three binning results and extracts the best bin set by comparing the completeness and contamination rates of each bin estimated with CheckM.^51^ The MetaWRAP result is summarized in Supplementary Figs S9 and S10. The comparison of the completeness and contamination rate of bins in MetaBAT2 and MetaWRAP indicates that MetaWRAP outperforms MetaBAT2 for the genome completeness of each bin. On the other hand, the contamination rate of MetaWRAP bins is sometimes higher than that of MetaBAT2. For example, the *Faecalibacterium prausnitzii* and *Agathobacter rectalis* genomes are highly contaminated in the MetaWRAP binning result of the NovaSeq sequencing data of the DNA-mix sample (Supplementary Figs S9 and S10). These results indicate that MetaBAT2 strictly divides bins to reduce the contamination possibility but often a single genome is divided into two or more bins. Thus, the current versions of either tool are not ideal for binning complex microbial communities and further tool development is necessary.

In conclusion, we have constructed a new well-balanced bacterial mock community representing the major taxa of the human gut bacterial community regardless of country/residential area, by flow cytometry-based quantification and strain-specific qPCR. Taking advantage of this mock community, we compared the performance of different experimental methods commonly used in human gut microbial studies. A detailed investigation based on the reference genomes revealed that PCR primers for 16S rRNA genes and DNA extraction methods have a substantial influence on the taxonomic compositions inferred from the sequenced reads. MetaBAT2-generated phylogenetically consistent bins although the completeness of the generated bins varied among the strains. Our comparative analyses provide an important baseline for human gut microbiome studies. As methodologies for human gut microbiome analysis progress, benchmark studies using mock communities like the one described here should continue to be conducted, and the data should be shared without restriction.

## Supplementary Material

dsad010_suppl_Supplementary_Figure

dsad010_suppl_Supplementary_Table
