# Supplementary material for: Assessment of metagenomic workflows using a newly constructed human gut microbiome mock community
Source: DNA Res. 2023 May 31;30(3):dsad010. doi: 10.1093/dnares/dsad010 (PMC10229288; doi:10.1093/dnares/dsad010)
Supplement: dsad010_suppl_Supplementary_Figure [file dsad010_suppl_supplementary_figure.pdf]

Supplementary methods

1. Gram staining

Gram staining was performed by using the neo-B&M (Wako Pure Chemical Industries, Osaka, Japan). Crystal violet was applied to the smears for 1 min, and slides were briefly rinsed under water to remove excess crystal violet. Gram iodine mordant was applied for 30 seconds and the slides were tilted to remove the reagents. Gram iodine mordant was applied again for 30 seconds. To remove any non-specific crystal violet staining, the slides were briefly rinsed by Gram decolorizer solvent (30% acetone and 70% ethanol) and rinsed under ultra-pure water. The sections were then counterstained with carbol fuchsin for 1 min, and then briefly rinsed under ultra-pure water.

2. DNA extraction methods

Bacterial strains were stored at -80°C until ready for use. DNA extraction of bacteria was performed according to the following 10 protocols. Manufacturers' instructions were followed for all methods except where noted.

#1 Enzymatic lysis

A sample suspended in 475 µl of TE buffer (pH8.0, FUJIFILM Wako Pure Chemical Corporation, Osaka, Japan) and 25 µl of lysozyme (300 mg/ml, FUJIFILM Wako pure Chemical Corporation, Osaka, Japan) was added to the suspension followed by incubation for 60 min at 37°C. After the addition of 55 µl of achromopeptidase (20,000U/ml, FUJIFILM Wako Pure Chemical Corporation, Osaka, Japan), the mixture was incubated for 30 min at 37°C. The samples were then added with 61.5 µl of 10% SDS (Nacalai tesque, Kyoto, Japan) and 25.7 µl of Proteinase K (25 mg/ml, Merck, Kenilworth, NJ, USA) followed by incubation for 60 min at 55°C. We then added 650 µl of mixture of phenol:chloroform:isoamyl alcohol (25:24:1, Nacalai tesque, Kyoto, Japan), and mixed gently for 5 min. After centrifugation at 13,000 rpm for 5 min at room temperature, collected all of supernatant fraction and repeated the above phenol-chloroform procedure. 1,000 µl of Ethanol (99.5%, FUJIFILM Wako Pure Chemical Corporation, Osaka, Japan) and 50 µl of sodium acetate (3M, Nacalai tesque, Kyoto, Japan) were added to collected supernatant and then left standing for 15 min at -30°C.

After centrifugation at 15,000 rpm for 10 min at 4°C, we discarded supernatant and added 1,000 µl of 70% ethanol to the pellet. After centrifugation at 15,000 rpm for 5 min at 4°C, we discarded supernatant and suspended in 300 µl TE buffer. DNA solution was added with 3 µl of Ribonuclease (DNase free) (10 mg/ ml, NIPPON GENE CO., LTD., Toyama, Japan) and incubated for 30 min at 37°C. After incubation, RNase treated sample was mixed 303 µl of 10% polyethylene glycol solution (Polyethylene Glycol #6000, Nacalai tesque, Kyoto, Japan). The mixture was incubated for 30 min at 4°C and washed with 75% EtOH. After centrifugation at 15,000 rpm for 5 min at 4°C, we removed the supernatant and dried up. DNA pellet was resuspended in 100 µl TE buffer.

#2 Beads Phenol

Procedure of this method was described by Matsuki et. al. [1]. Two hundred and fifty µl of the Extraction buffer (200 mM Tris-HCl, 80 mM EDTA, pH 9.0), 50 µl of 10% SDS, 0.3 g of glass beads (φ 0.1 mm, BioSpec Products Inc., Bartlesville, OK), and 500 µl of TE Saturated Phenol (NIPPON GENE CO., LTD., Toyama, Japan) were added to a sample tube containing a cell mixture. The mixture was disrupted with glass beads by vigorous shaking (1500 rpm, for 30 sec) using a Shake Master (Biomedical Science, Tokyo, Japan), and then centrifuged at 14,000 rpm for 5 min. 400 µl of supernatant was transferred to a new tube, and 400 µl of phenol/chloroform/isoamyl alcohol (25:24:1, Nacalai tesque, Kyoto, Japan) was added. Then, the mixture was mixed by inverting the tube for 45 sec. After centrifugation at 14,000 rpm for 5 min, 250 µl of supernatant was transferred to a new tube. 25 µl of 3 M sodium acetate (Nacalai tesque, Kyoto, Japan) and 250 µl 2-propanol (Nacalai tesque, Kyoto, Japan) were added and briefly mixed. After centrifugation at 14,000 rpm for 5 min, the supernatant was removed, and the remaining pellet was washed with 500 µl of 70% ethanol to remove 2-propanol. After centrifugation at 14,000 rpm for 5 min, the supernatant was discarded, and the pellet was dried and resuspended in 100 µl of TE buffer.

#3 MetaHIT method

This protocol was described by Qin et. al. [2]. Two hundred and fifty µl of guanidine thiocyanate (Guanidine thiocyanate for molecular biology, Sigma-Aldrich Co. LLC, St.

Louis, USA), 40 µl of 10% N-lauryl sarcosine (Sigma-Aldrich Co. LLC, St. Louis, USA), and 500 µl of 5% N-lauryl sarcosine were added to a cell mixture, and the mixture was centrifuged briefly. After the heat treatment at 70°C for 1 hour, 750 mg of zirconia/silica beads (φ 0.1 mm, Biospec Products Inc., Bartlesville, OK) was added. The mixture was disrupted in the following order; 5 min of bead-beating, 10 min rest, and bead-beating again for 5 min. Fifteen mg of poly(vinylpolypyrrolidone) (PVPP, Sigma-Aldrich Co. LLC, St. Louis, USA) was added and centrifuged at 14,000 rpm for 5 min at 4°C. The supernatant was transferred to a new tube and remained pellet was resuspended in 500 µL of TENP (50 mM Tris (pH 8.0), 20 mM EDTA (pH 8.0), 100 mM NaCl, and 1% PVPP). After centrifugation at 14,000 rpm for 5 min at 4°C, the new supernatant was added to the first supernatant. This TENP procedure was repeated three times. The pooled supernatant was dispensed to two new 2 ml tubes, and 1 ml of 2-propanol was added to each tube. After incubation for 15 min at room temperature, tubes were centrifuged at 14,000 rpm for 10 min at 4°C. The pellets were dried and resuspended in 450 µl of 100 mM phosphate buffer (pH 8.0) and 50 µl of 5 M potassium acetate (FUJIFILM Wako Pure Chemical Corporation, Osaka, Japan). Tubes were placed on ice for 90 min and then centrifuged at 14,000 rpm for 30 min at 4°C. The supernatant was transferred to a new 2 ml tube and added 2µl of RNase (10 mg/ ml, NIPPON GENE CO., LTD., Toyama, Japan). The tube was incubated for 30 min at 37°C, then placed on a -20°C freezer for overnight after addition of 50 µl of 3 M sodium acetate and 1 ml of 100% ethanol. After centrifugation at 14,000 rpm for 30 min at 4°C, the supernatant was removed and 1 ml of 70% ethanol was added to the pellet. After centrifugation at 14,000 rpm for 5 min at 4°C, the supernatant was removed and 500 µl of 70% ethanol was added to the pellet. Finally, after centrifugation at 14,000 rpm for 5 min at 4 °C, the pellet was resuspended in 100 µl of TE buffer after drying.

#4 HMP method

This protocol was described by Human Microbiome Project [3]. The DNeasy PowerSoil kit (Qiagen, Hilden, Germany) was used according to the procedure of manufacturer's instruction with a few modification as follows. A cell mixture and 60 µl of solution C1 were added to the PowerBead tube. The tube was vortexed for 10 min at the maximum

speed at room temperature. After centrifugation at 10,000 g for 30 sec at room temperature, the supernatant was transferred to a clean new tube. Two hundred and fifty µl of solution C2 was added and then the cell mixture was incubated at 4°C for 5 min. After centrifugation at 10,000 g for 1 min, 600 µl of supernatant and 200 µL of solution C3 were mixed and incubated at 4°C for 5 min. After centrifugation at 10,000 g for 1 min, 750 µL of supernatant and 1200 µL of the solution C4 were mixed, and the mixture was passed through the MB spin column by 10,000 g for 1 min centrifugation. Column was washed with the solution C5, and finally 100 µL of TE buffer was added to the column and centrifuged at 10,000 g for 30 sec to elute the DNA.

#5 QIAamp

QIAamp DNA Stool kit (Qiagen, Hilden, Germany) was used exactly according to the procedure of manufacturer's instruction.

#6 NucleoSpin

The NucleoSpin® DNA Stool (Takara Bio, Shiga, Japan) was used exactly according to the procedure of manufacturer's instruction.

#7 PureLink

The PureLink Microbiome DNA Purification Kit (Thermo Fisher Scientific, Waltham, MA, USA) was used exactly according to the procedure of manufacturer's instruction.

#8 MonoFas

The MonoFas DNA Stool Extraction Kit X (GL Sciences, Saitama, Japan) was used exactly according to the procedure of manufacturer's instruction.

#9 Zymo Quick-DNA

The Quick-DNA Fecal/Soil Microbe Mini Kit (Zymo Research, Orange, CA, USA) was used exactly according to the procedure of manufacturer's instruction.

#10 FastDNA

FastDNA SPIN Kit for Soil (MP Biomedicals, Solon, OH, USA) was used exactly

according to the procedure of manufacturer's instruction.

To compare and assess the differences among the 10 DNA extraction methods described above, the extracted microbial DNAs by #5 to #10 DNA extraction kits were eluted with 100 µl of TE buffer. Concentrations of the extracted DNA were determined fluorometrically (Qubit® dsDNA BR assay, Life Technologies Europe, Naerum, Denmark). The purity of the DNA solutions was measured spectrophotometrically (NanoDrop 1000 Spectophotometer, Thermo Fisher Scientific, Waltham, MA, USA).

3. Quantitative real-time PCR (qPCR) measurement

The threshold cycle (Ct) value for qPCR was calculated by the 2nd Derivative Maximum (SDM) method using a Thermal Cycler Dice Real Time System TP800 (Takara Bio, Shiga, Japan). The composition of the reaction mixture (25 µL) was 0.5 µL of forward primer (10 mM), 0.5 µL of reverse primer (10 mM), 9.5 µL of UltraPure DNase/RNase-Free Distilled Water (Thermo Fisher Scientific, Waltham, MA, USA), 12.5 µL of TB Green Premix Ex Taq II (Tli RNase H Plus) (Takara Bio, Shiga, Japan), and 2.0 µL of extracted DNA as template. PCR conditions suitable for each strain-specific *rpoB* PCR primer pair were determined and used in this study. Amplification efficiency was calculated as follows. DNA was extracted from 20 µL of a single strain solution and serially diluted in TE at 10^1, 10^2, 10^3 and 10^4 times. The obtained DNA solutions were subjected to qPCR in duplicates, and the PCR amplification efficiency (e) was calculated from the slope of the linear regression (s) between the qPCR Ct value and the dilution rate as e=(10^(-1/s))-1. Abundance estimation of each taxon from the DNA mix sample was performed independently per taxon by the qPCR quantification using each strain-specific *rpoB* PCR primer pair. The relative abundance of each taxon in 18 taxa was calculated using these abundance estimation results.

For electrophoresis of qPCR products, 3.0% (w/v) agarose gel (NIPPON Genetics, Tokyo, Japan) with 1 mg/mL of ethidium bromide (Bio-Rad Laboratories, Hercules, CA, USA) was used. One µL of 6 × Loading Dye (TOYOBO, Osaka, Japan) was added to 5 µL of the qPCR product solutions. Three µL of 100 bp DNA Ladder H3 RTU DNA marker (NIPPON Genetics, Tokyo, Japan) was used to estimate the product lengths. Electrophoresis gels were observed with UV light to obtain the images.

4. 16S rRNA gene amplification and amplicon sequencing

The first PCR reaction mixture contained 1X KAPA HiFi HotStart ReadyMix (Kapa biosystems, Wilmington, MA, USA), each target-specific primer set tailed with forward and reverse universal sequences (0.2 μM each), and 1 ng of genomic DNA in a total volume of 25 μL per sample. PCR amplification conditions were as follows: an initial denaturation step at 95°C for 3 min, 25 denaturation cycles at 95°C for 30 sec, annealing (50°C for 515F-806R, 515FY-806RN and 515FY-926RY, 54°C for 968F-1390R, and 55°C for 27Fmod-338R, 341F-785R and 342F-806RS) for 30 sec, extension step at 72°C for 30 sec, and a final extension at 72°C for 5 min. PCR products were purified with Agencourt AMPure XP (Beckman Coulter, Brea, CA, USA) and eluted in 50 μL 10mM Tris (pH 8.5) solution. The second PCR amplification was performed for 8 cycles using 1X KAPA HiFi HotStart ReadyMix (Kapa biosystems, Wilmington, MA, USA), 2.5 μL of each indexing PCR primer in the Nextera XT Index Kit (Illumina, San Diego, CA, USA) and 2.5 μL of purified PCR products in a 25 μL reaction mixture per sample.

Amplicon sequence analysis by DADA2 was conducted with the following parameters: filterAndTrim with maxN = 0, maxEE = c(4,6) or maxEE = 4 for the three single-end samples, truncQ=2, trimLeft = 28, trimRight = 28, minLen = 150, rm.phix = TRUE, rm.lowcomplex = TRUE, and removeBimeraDenovo with the consensus method

5. Inference of strain composition based on shotgun metagenomic sequences

(i) Read mapping on the reference genomes ("Mapping"): this method infers taxonomic abundance by conducting DNA sequence similarity searches of short reads against reference genomes. Forward short reads were mapped on the RefSeq genomes of the 18 strains using bowtie2 version 2.3.5.1. The reads that uniquely mapped to a CDS region were enumerated using htseq-count version 0.11.2 with the union mode.

(ii) Read mapping on the de novo assembled contigs/scaffolds ("Assembly"): this method infers taxonomic abundance by mapping the short reads on the contigs and scaffolds generated by MEGAHIT version 1.2.9 and SPAdes version 3.13.1 with the metagenomic mode, respectively. CDSs within contigs/scaffolds were predicted using Prodigal version 2.6.3, and those which had unique and identical hits (alignment length ≥ 35 amino acids) with the reference protein sequences of the CDSs of the 18 strains were

taken into account. Mapping of short reads on the contigs and enumeration of the reads mapped on the CDS regions were conducted as described in the (i) Mapping procedure.

(iii) Binning: this method infers taxonomic abundance by counting the mapped short reads on the not whole contigs/scaffolds but those clustered into the bins by MetaBAT2 version 2.14. A bin and the source strain were associated based on the RefSeq protein IDs of the CDSs assigned in the (ii) Assembly procedure. If a bin consisted of contigs derived from multiple strains, the strain of which RefSeq protein IDs were most highly represented was selected as the source strain of that bin. We then estimated the whole-genome completeness of each bin based on the ratio of total length of binned contigs to the genome size of the source strain. The bin boasting the highest completeness within each strain was selected as the "representative bin" of that strain, and we enumerated the reads mapped on the CDS regions within contigs of the representative bins. In addition to completeness, we estimated the whole-genome contamination rates of bins based on the ratio of the number of RefSeq protein IDs which were not derived from the source strain to that of all RefSeq protein IDs retrieved in a bin. Besides our manual estimation of the completeness and contamination rates on the whole-genome scale, the completeness and contamination rates of bins were also estimated by using CheckM version 1.0.12, based on the presence or absence of 104 *Bacteria*-conserved single-copy marker gene sets [4]. MetaWRAP version 1.3.2 was also conducted for binning of assembled contig/scaffolds. Within MetaWRAP, three binning tools (MetaBAT2 version 2.14, MaxBin version 2.2.7, CONCOCT version 1.1.0) were used for binning.

(iv) CDS prediction from short reads ("ReadCDS"): this method infers taxonomic abundance by conducting protein sequence deduced from the unassembled short reads. Protein sequences of CDSs were predicted from forward short reads using Prodigal version 2.6.3 with the metagenomic mode. Predicted partial CDS sequences (ReadCDS) were then subjected to BLASTP search against the reference protein sequence of 18 strains with the criteria described in the (ii) Assembly procedure.

References

1. Matsuki, T., Watanabe, K., Fujimoto, J., et al. 2004, Quantitative PCR with 16S

rRNA-gene-targeted species-specific primers for analysis of human intestinal bifidobacteria, Appl. Environ. Microbiol., 70, 167-173.

2. Qin, J., Li, R., Raes, J., et al. 2010, A human gut microbial gene catalogue established by metagenomic sequencing, Nature, 464, 59-65.
3. Human Microbiome Project Consortium. 2012, Structure, function and diversity of the healthy human microbiome, Nature, 486, 207-214.
4. Parks, D.H., Imelfort, M., Skennerton, C.T., Hugenholtz, P., and Tyson, G.W. 2015, CheckM: assessing the quality of microbial genomes recovered from isolates, single cells, and metagenomes, Genome Res., 25, 1043-1055.

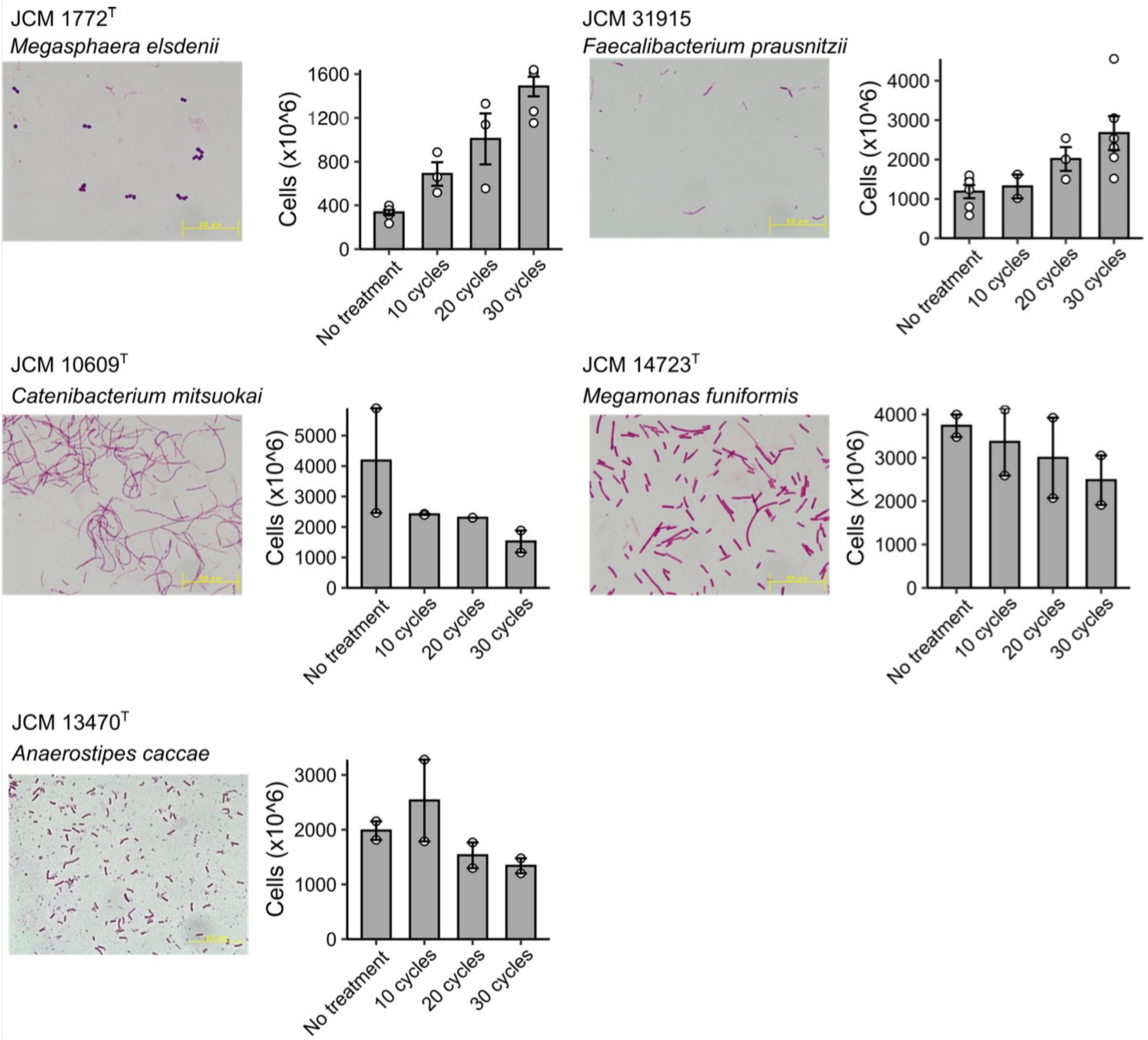

Figure S1. Impact of ultrasonication on the bacterial cell counts.
Gram staining images of each strain after ultrasonic treatment (left) and cell counts with flow cytometry using a bacteria counting kit, as described in methods, of the cell suspensions treated with different sonication conditions (right) of the representative strains. Ultrasonic treatment was performed at 68 kHz for 20 sec, and for 10, 20 or 30 cycles.

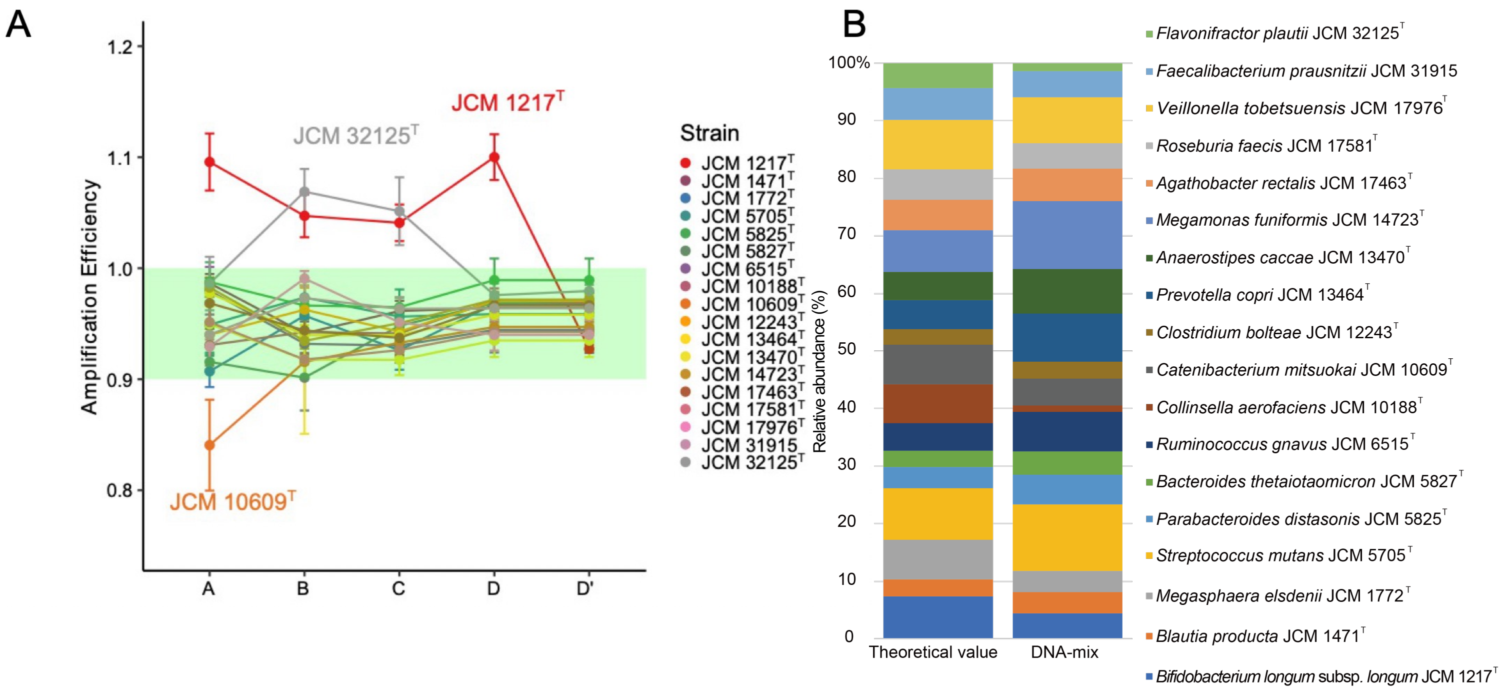

Figure S2. Strain-specific quantification by qPCR of *rpoB* genes. (A) PCR conditions (details: Table S8, condition A, B, C, D and D') using strain-specific *rpoB* primers were evaluated. The amplification efficiency calculation method is described in Supplementary Methods. (B) Comparison between theoretical value and DNA mix. The proportion of each taxon in the DNA mix sample was estimated from the results of the qPCR experiments using each strain-specific *rpoB* PCR primer. The theoretical value was estimated from the genome size and calculated as the ratio detected when the DNA amounts were equal.

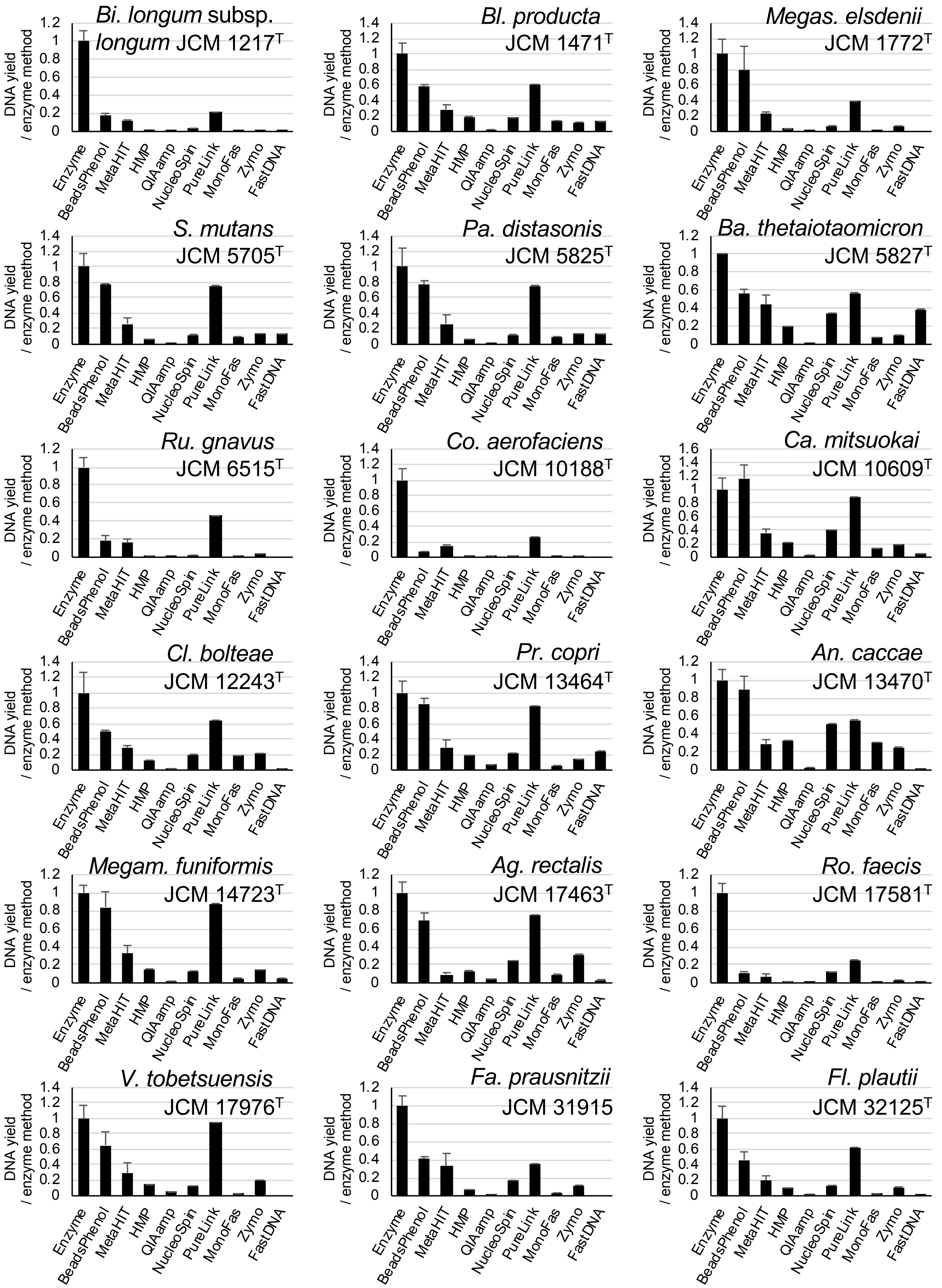

Figure S3. DNA yields from cultures of each of the 18 strains using different DNA extraction methods. The ratio of the amount of extracted DNA using each method to that of the enzyme method was calculated. For the Enzyme, BeadsPhenol and MetaHIT method, DNA was independently extracted four times. The other DNA extraction methods using commercial kits were performed twice. Error bars represent the standard error of the mean.

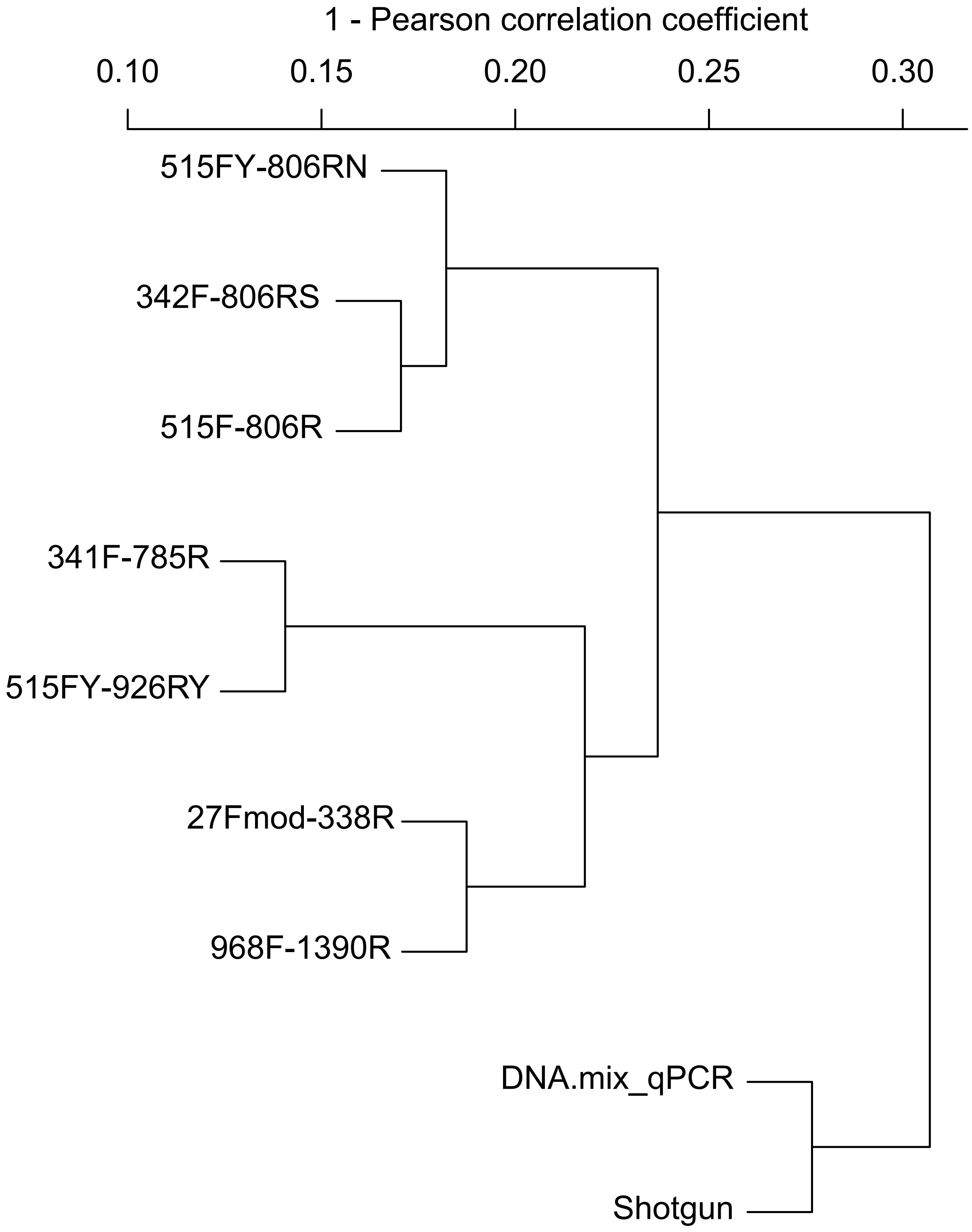

Figure S4. Hierarchical clustering result of strain compositions inferred by MAPseq. The strain compositions of seven amplicon sequencing using MAPseq, the strain composition of shotgun metagenomic sequencing of the DNA-mix sample, and the strain composition of qPCR quantifications of the DNA-mix sample were compared. Dissimilarity of strain compositions were calculated as 1 – Pearson correlation coefficient. Complete-linkage clustering was performed.

	MEGAHIT								SPAdes							
	DNA mix		Cell mix						DNA mix		Cell mix					
Completeness (%)	HiSeq	NovaSeq	Enzyme	HMP	BeadsPhenol	MetaHIT	PureLink	Zymo	HiSeq	NovaSeq	Enzyme	HMP	BeadsPhenol	MetaHIT	PureLink	Zymo
Bacteroides thetaiotaomicron	97.02	94.28	90.02	88.33	94.99	85.90	96.85	95.48	99.52	98.64	97.22	97.39	97.30	98.32	98.10	97.33
Parabacteroides distasonis	0	92.02	0	92.05	55.01	0	93.19	92.07	94.35	93.58	93.83	93.74	94.01	93.49	93.38	93.93
Agathobacter rectalis	50.25	38.78	38.84	44.79	52.85	39.91	36.06	28.34	81.09	45.82	45.87	45.76	45.86	44.30	61.34	45.86
Anaerostipes caccae	42.37	22.49	42.36	30.67	34.15	64.85	22.49	35.99	19.77	19.78	19.77	18.59	19.77	19.77	19.77	19.77
Prevotella copri JCM	43.02	34.08	27.56	21.04	22.02	30.15	32.81	24.02	90.11	39.73	36.20	32.05	26.62	31.77	41.15	20.27
Collinsella aerofaciens	51.82	33.53	56.77	85.25	81.31	85.94	53.11	55.96	90.98	62.06	91.11	84.31	90.58	90.54	89.55	90.82
Catenibacterium mitsuokai	77.47	48.31	36.65	40.75	42.40	40.09	46.55	48.45	73.42	64.04	35.21	50.06	42.31	53.79	57.60	52.72
Bifidobacterium longum	90.72	50.25	50.25	89.70	79.36	72.75	58.71	79.37	58.76	41.51	41.53	50.13	50.17	50.12	50.12	75.58
Flavonifractor plautii	72.20	64.75	81.94	68.89	49.24	63.00	42.01	77.96	83.35	50.63	67.03	60.34	85.21	63.30	83.37	85.48
Megamonas funiformis	35.58	24.45	24.72	24.45	38.03	32.57	32.58	34.60	54.60	35.18	31.78	32.24	41.18	29.19	38.65	52.42
Blautia producta	66.59	38.87	42.45	35.23	39.81	41.47	49.44	45.25	92.23	78.70	46.68	42.55	77.04	29.82	77.29	73.41
Veillonella tobetsuensis	93.94	93.16	93.36	89.91	61.94	93.36	93.32	91.99	94.08	58.52	45.76	54.14	45.74	93.85	93.86	94.08
Roseburia faecis	89.78	34.11	87.61	86.41	84.86	86.53	87.28	85.17	90.78	62.28	53.60	90.82	91.69	93.20	90.58	64.46
Clostridium bolteae	80.49	50.85	63.62	41.23	30.54	63.05	37.34	27.15	90.29	66.82	41.04	55.00	49.91	52.53	55.19	55.08
Faecalibacterium prausnitzii	40.02	34.07	40.01	42.10	40.47	40.01	41.58	40.00	36.44	38.48	47.64	33.25	33.37	33.20	33.26	41.89
Megasphaera elsdenii	46.07	24.98	31.21	15.15	57.57	63.59	26.39	50.23	73.58	54.94	48.96	45.74	54.95	49.16	45.73	45.74
Streptococcus mutans	57.22	72.19	43.90	53.49	72.77	49.00	50.63	44.91	98.37	71.31	70.16	98.38	98.37	71.44	98.37	70.20
Ruminococcus gnavus	40.21	26.16	37.66	28.94	39.40	45.45	37.23	30.48	50.66	23.56	46.04	34.51	49.91	64.46	72.70	46.87
Contamination (%)																
Bacteroides thetaiotaomicron	0.02	0.02	0.05	0	0.05	0	0.32	0	2.10	0.53	0.09	0.02	0	0.13	0.09	0.09
Parabacteroides distasonis	0	0	0	0	0	0	0	0	0	0	0	0	0	0	0	0
Agathobacter rectalis	0.07	3.73	0.09	0	0	0.09	0	0	0	0	0	0	0	0	0	0
Anaerostipes caccae	0	0	0	0	0	0	0	0	0	0	0	0	0	0	0	0
Prevotella copri	0	0	0	0	0	0	0	0	0	0	0	0	0	0	0	0
Collinsella aerofaciens	0	0	0	0	0	0	0	0	0	0	0	0	0	0	0	0
Catenibacterium mitsuokai	0	0	0	0	0	0	0	0	0	0	0	0	0	0	0	0
Bifidobacterium longum	0	0	0	0	0	0	0	0	0	0	0	0	0	0	0	0
Flavonifractor plautii	0	0	0	0	0	0	0	0	0	0	0	0	0	0	0	0
Megamonas funiformis	0	0	0	0	0	0	0	0	0	0	0	0	0	0	0	0
Blautia producta	0.03	0	0	0	0	0	0	0	1.93	0	3.82	0	0	0.27	6.70	1.76
Veillonella tobetsuensis	0	0	0	0	0	0	0	0	0	0	0	0	0	0	0	0
Roseburia faecis	3.17	0	0	0	0	0	0	0	0.15	0.44	0	0.08	0	0.07	0.08	0
Clostridium bolteae	0	0	0	0	0	0	0	0	0	0	0.04	0	0	0.03	0.03	0.03
Faecalibacterium prausnitzii	0	0	0	1.97	0.09	0	0	0	0	0	0	0	0	0	0	0
Megasphaera elsdenii	0	0	0	0	0	0	0	0	0	0	0	0	0	0	0	0
Streptococcus mutans	0	0	0	0	0	0	0	0	0	0	0	0	0	0.23	0	0
Ruminococcus gnavus	0.08	0	0	0.11	0	0.07	0	0.10	4.40	0	0	0	4.68	0.05	3.25	0.07

Figure S5. Completeness and contamination rate of the representative bin for each strain from all assemblies.

Red and blue columns indicate high and low completeness rates, respectively. Green columns indicate relatively high contamination rates.

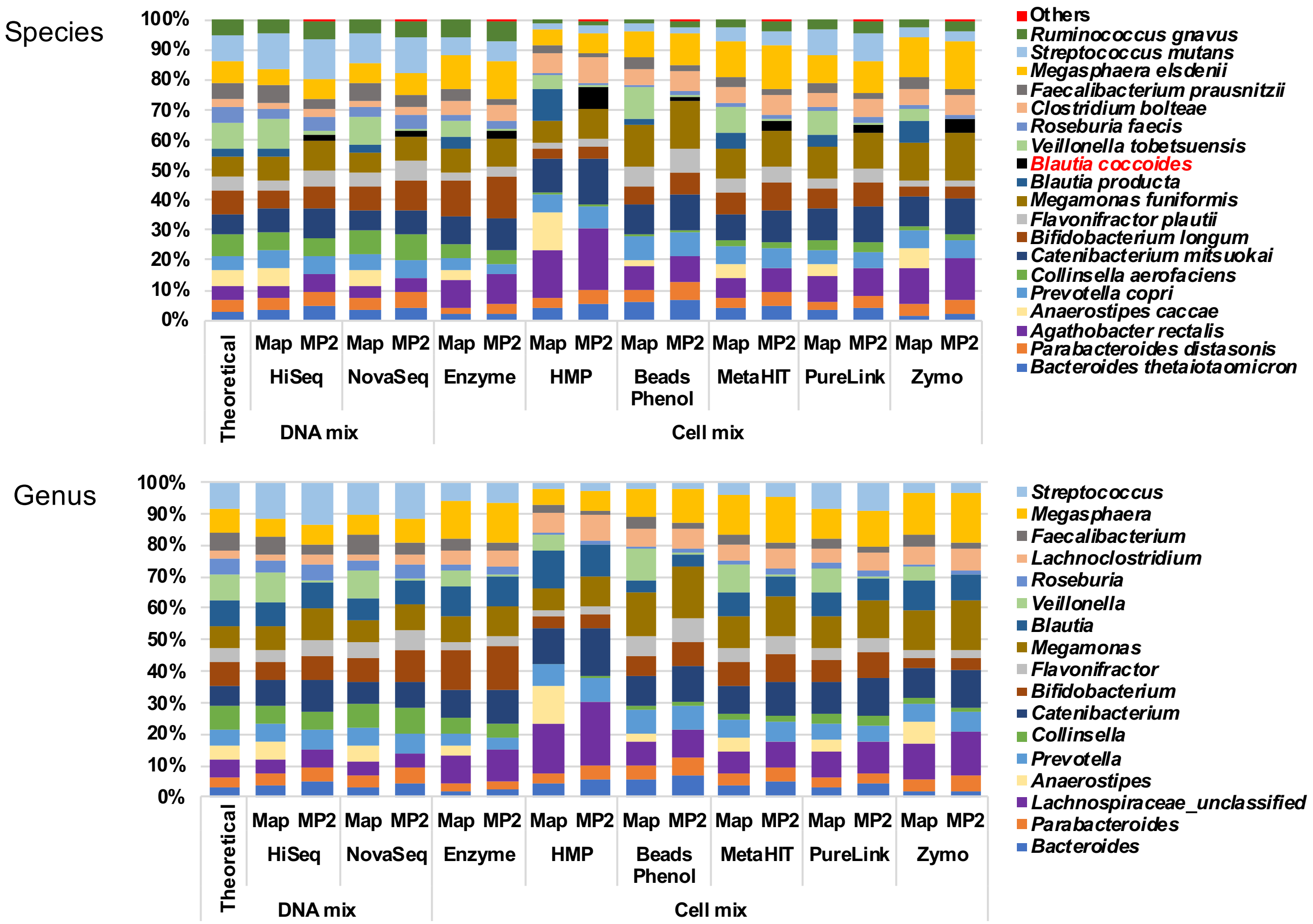

Figure S6. Taxonomic compositions estimated by the mapping procedure (Map) and MetaPhlAn2 pipeline (MP2). Genus classification is according to MetaPhlAn2, thus reads of *R. gnavus* are included in *Blautia* at the genus level. Unlike as shown in Fig. 5, compositions of the DNA-mix samples were not adjusted based on genome sizes. Instead, theoretical compositions for the DNA-mix sample without genome-size adjustment is indicated.

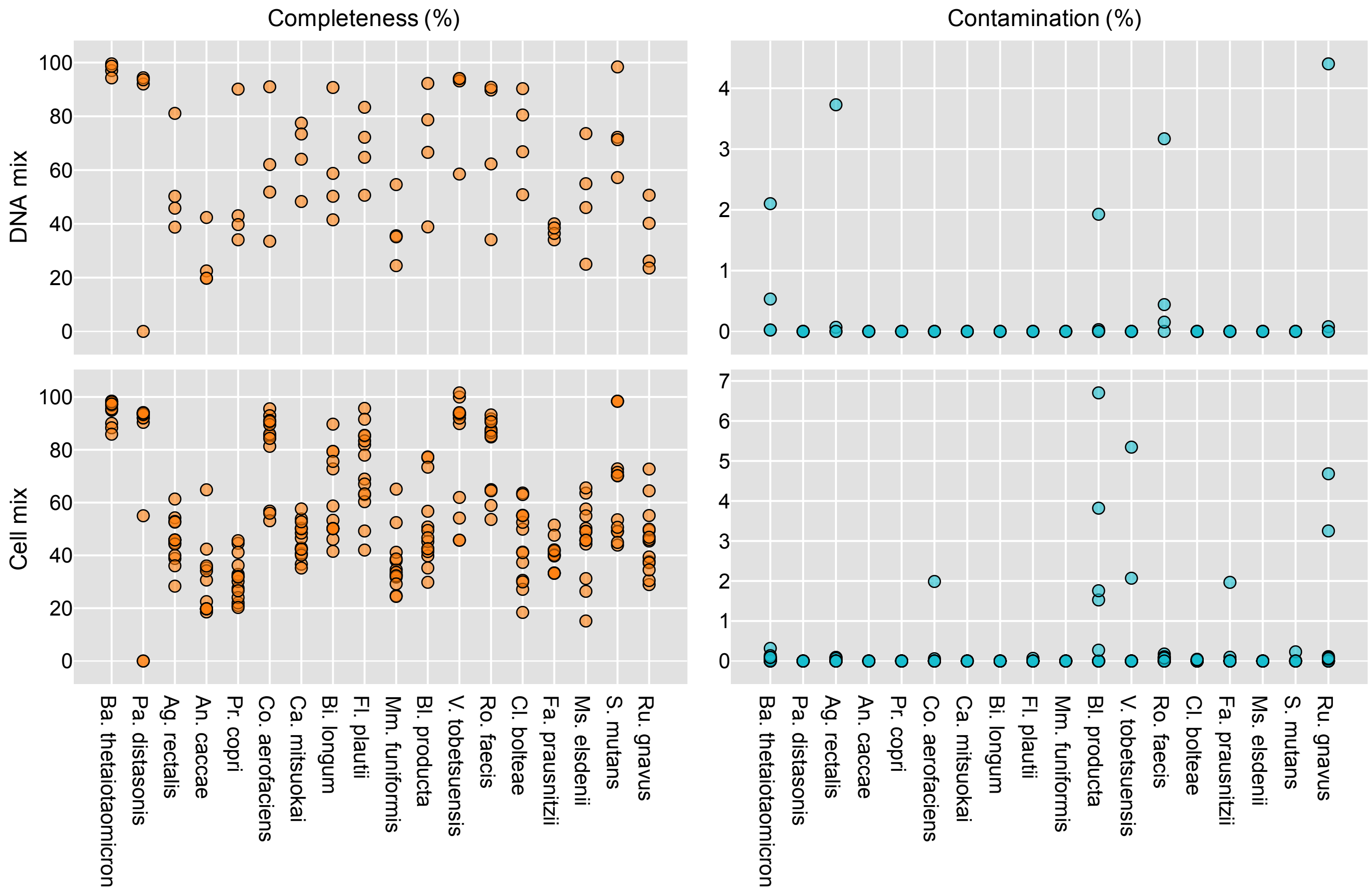

Figure S7. Distribution of the completeness and contamination rates of the representative bins obtained from the single-sample assemblies.

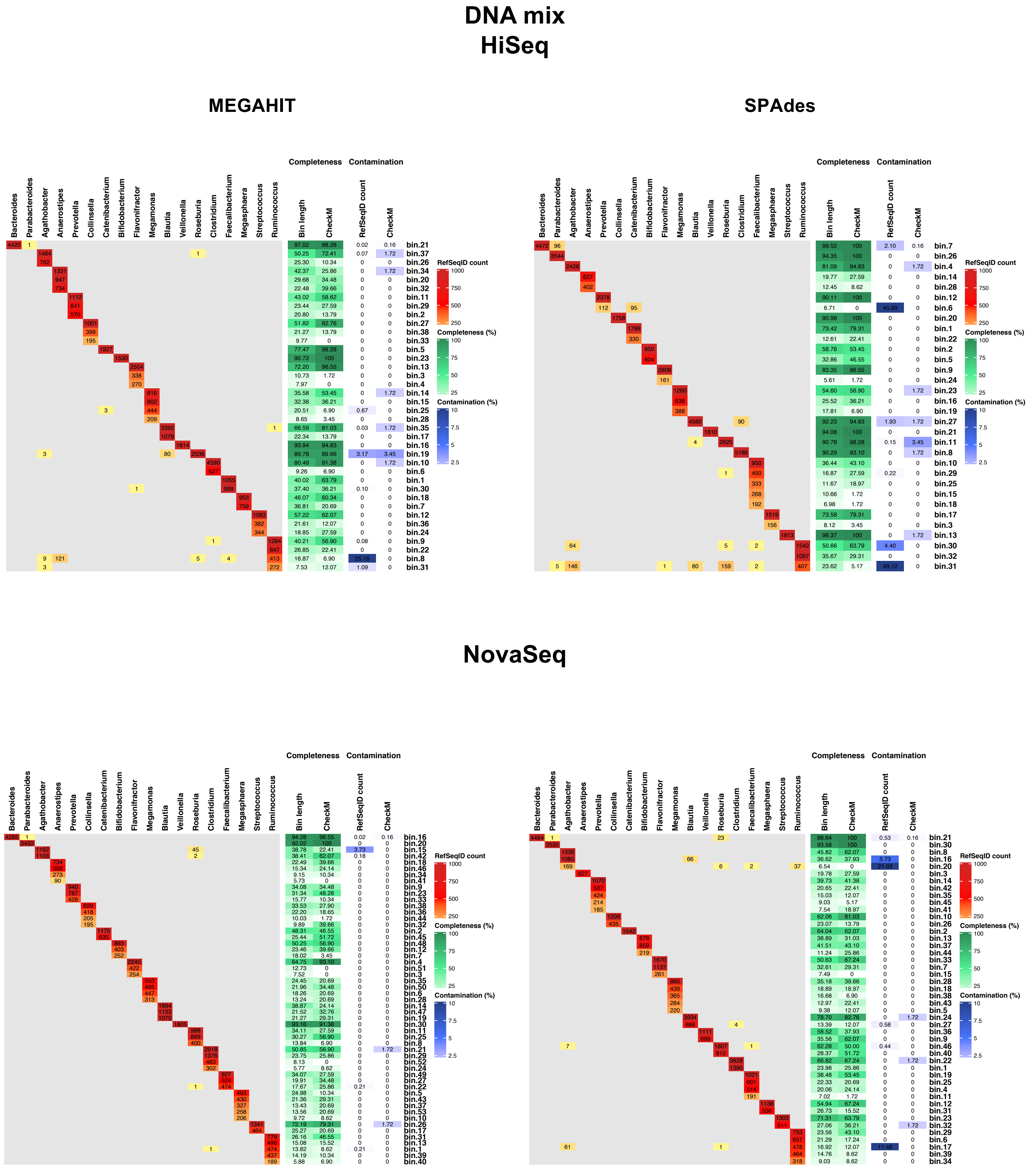

Figure S8. Distribution of RefSeq protein IDs among all bins reconstructed by MetaBAT2.
Completeness and contamination rate of a bin calculated by our manual procedures and CheckM are also shown.

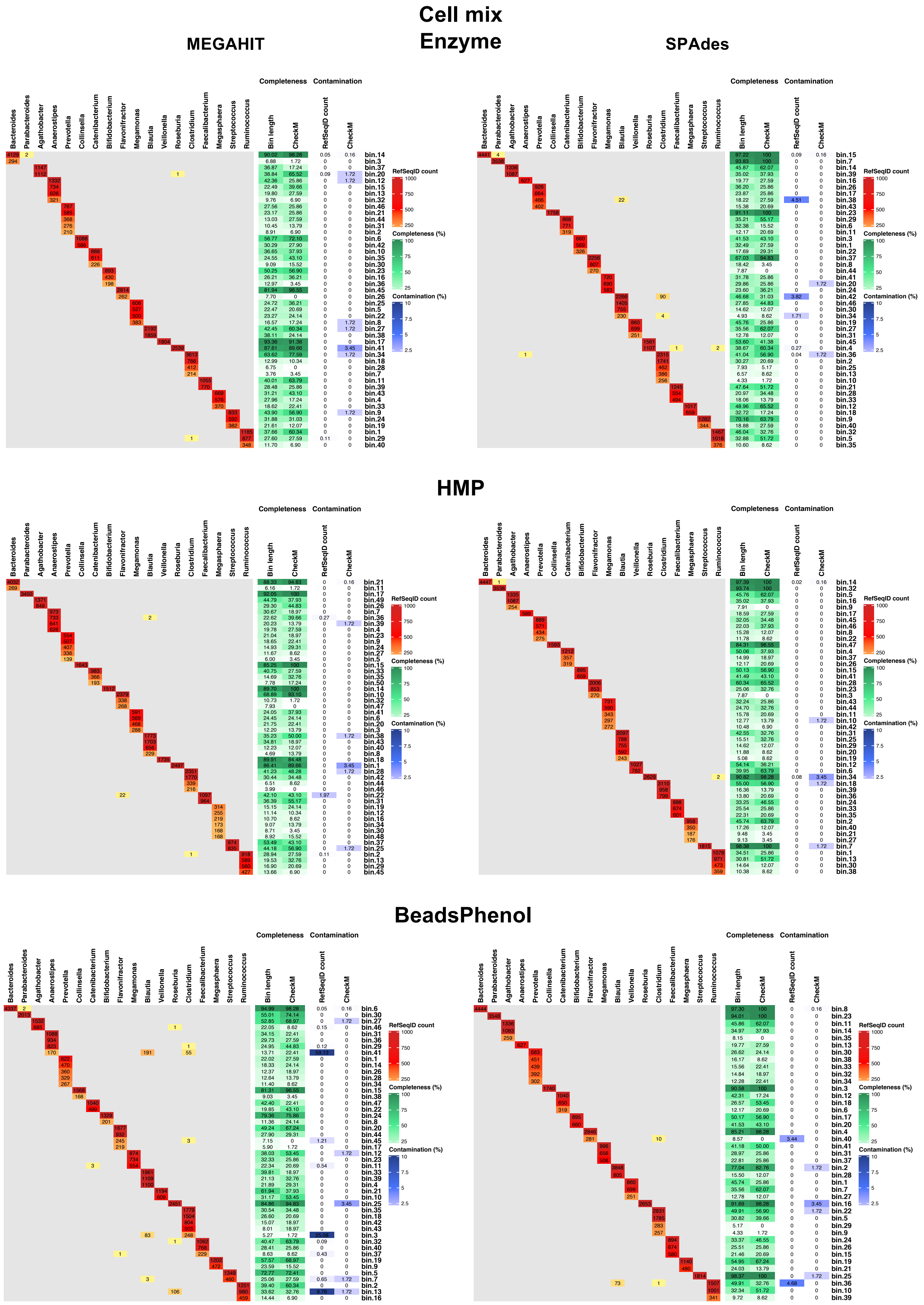

Figure S8. Continued

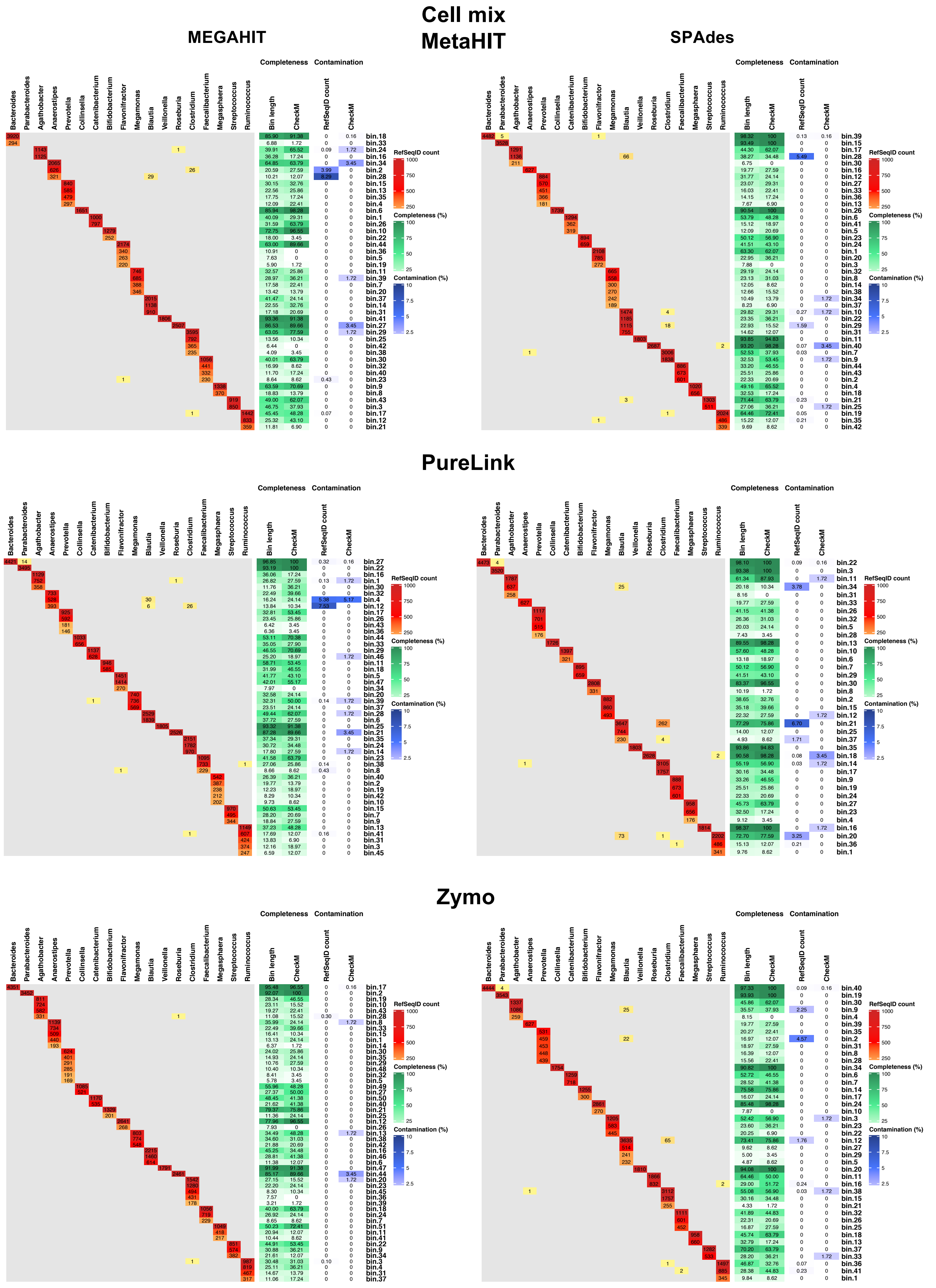

Figure S8. Continued

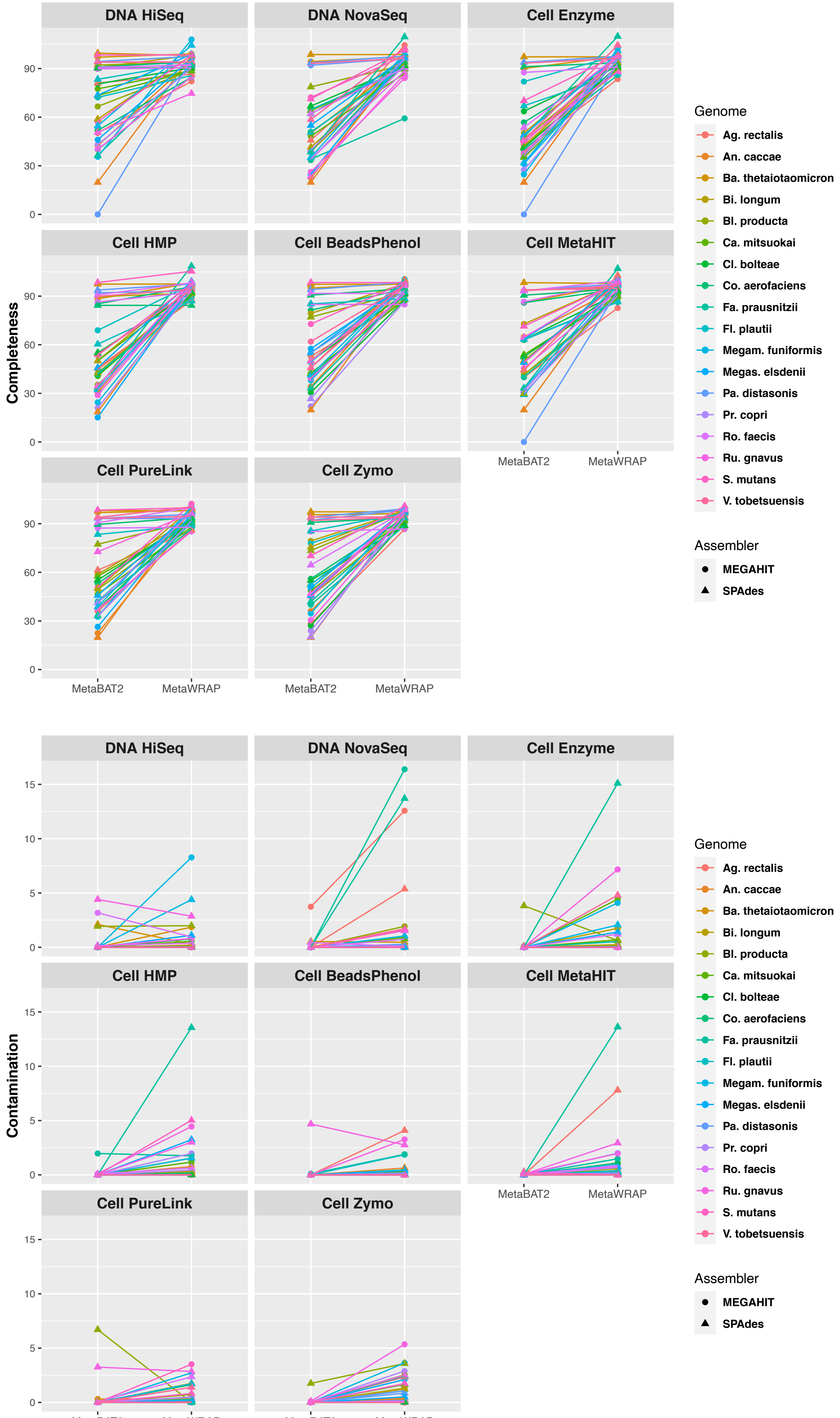

Figure S9. Comparison of bin quality between MetaBAT2 and MetaWRAP.

DNA mix

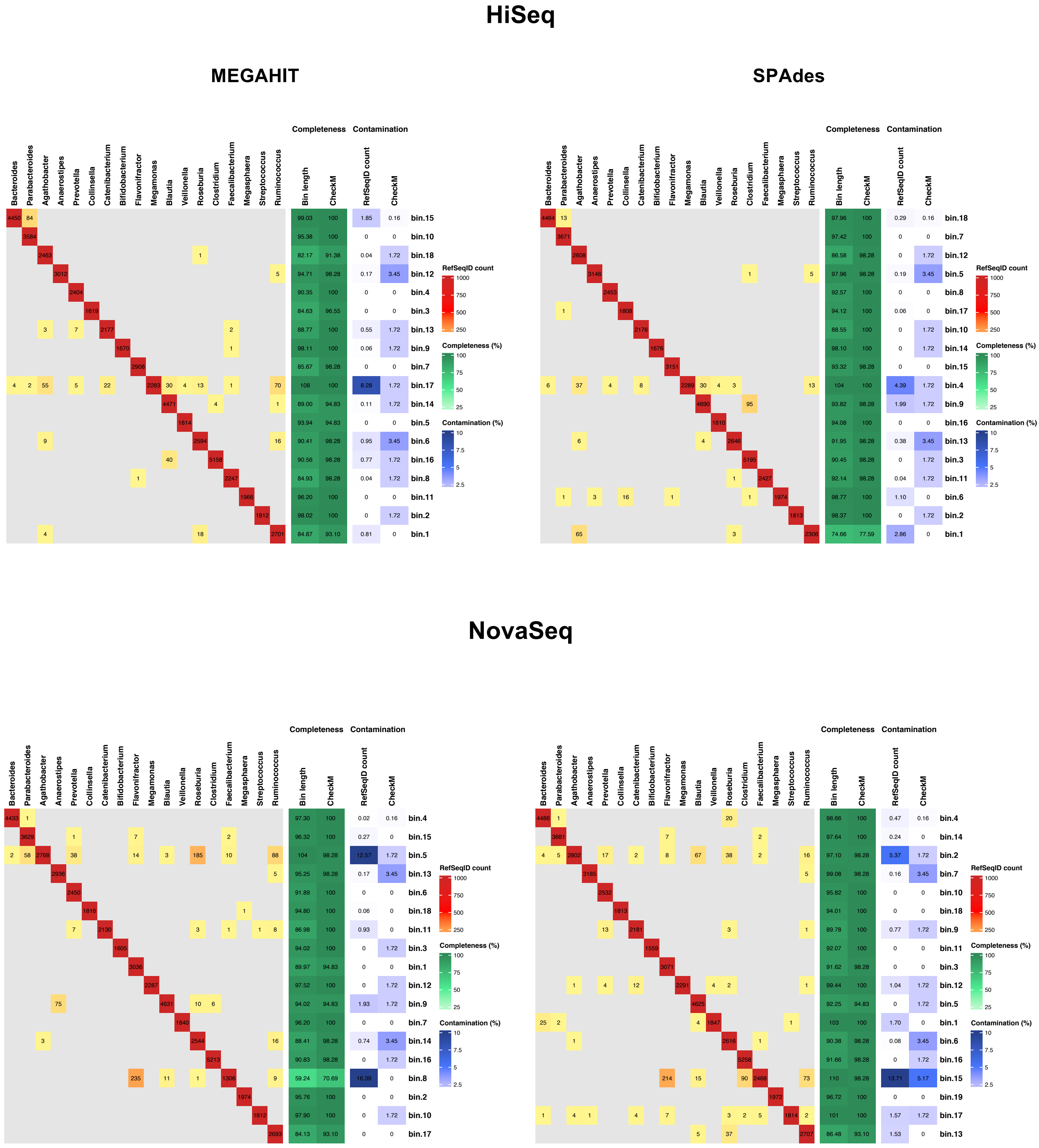

Figure S10. Distribution of RefSeq protein IDs among all bins reconstructed by MetaWRAP.
Completeness and contamination rate of a bin calculated by our manual procedures and CheckM are also shown.

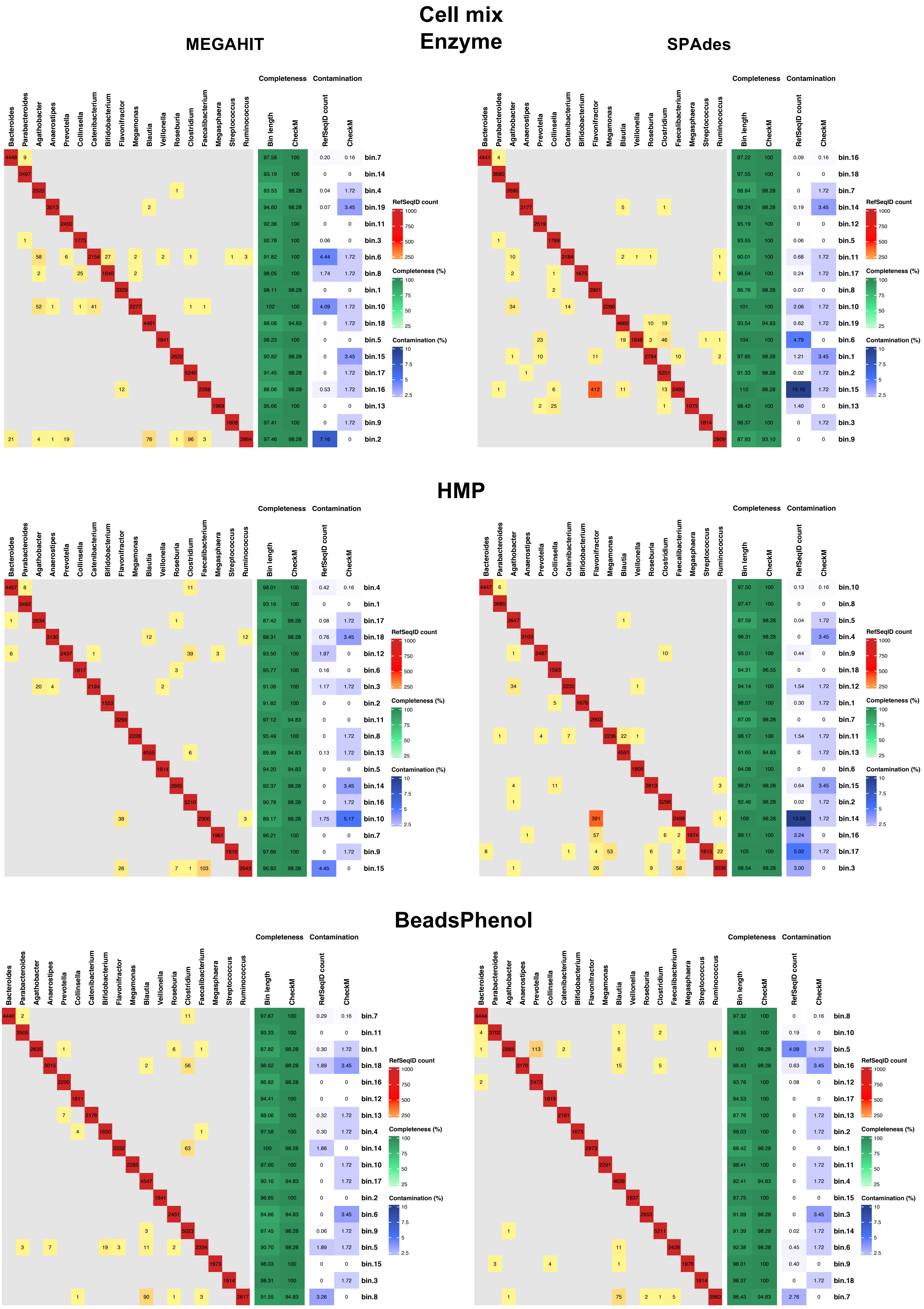

Figure S10. Continued

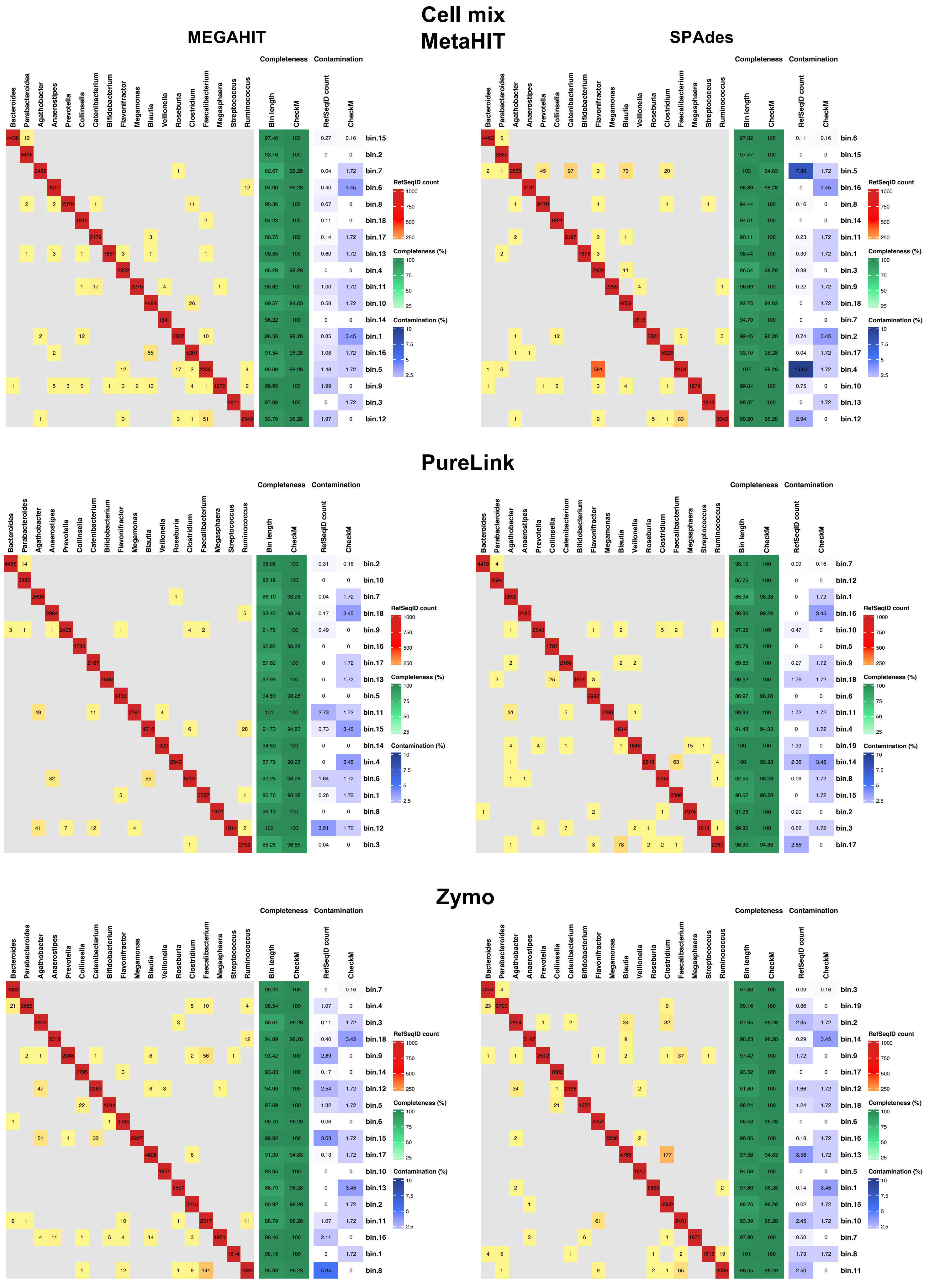

Figure S10. Continued
